# Integrin‐Piezo1 Axis Drives ECM Remodeling and Invasion of 3D Breast Epithelium

**DOI:** 10.1002/advs.202509932

**Published:** 2025-10-13

**Authors:** Kabilan Sakthivel, Anna Kotowska, Zhimeng Fan, Ellen Juel Portner, Catherine Merry, Pontus Nordenfelt, Adam Cohen Simonsen, Amanda J. Wright, Vinay S Swaminathan

**Affiliations:** ^1^ Division of Oncology Department of Clinical Sciences Lund University Lund 22100 Sweden; ^2^ Wallenberg Centre for Molecular Medicine Lund University Lund 22100 Sweden; ^3^ Optics and Photonics Research Group Faculty of Engineering University of Nottingham Nottingham NG72RD UK; ^4^ Department of Physics Chemistry and Pharmacy University of Southern Denmark Odense 5230 Denmark; ^5^ Division of Infection Medicine Department of Clinical Sciences Lund University Lund 22100 Sweden; ^6^ Biodiscovery Institute, Faculty of Medicine and Health Sciences University of Nottingham Nottingham NG72RD UK; ^7^ Department of Medical Biochemistry and Microbiology Uppsala University Uppsala 75237 Sweden

**Keywords:** 3D Mechanotransduction, ECM remodeling, Integrin signaling, Mammary acini, Stretch activated channels

## Abstract

Stiffening of tissue is a hallmark of cancer progression, promoting invasive phenotypes through altered cell‐extracellular matrix (ECM) interactions. However, how fully formed epithelial structures respond to mechanical cues within their native ECM environment remains poorly understood. Here, using a 3D in situ stiffening hydrogel system that enables modulation of stiffness around mature normal mammary acini, it uncovers critical steps in ECM remodeling and invasion of epithelial structures and discover molecular mechanisms driving this process. Stiffening around mature acini triggers two temporally distinct phases of epithelial remodeling, a rapid priming phase involving basement membrane (laminin, LN) disruption and fibronectin (FN) secretion, followed by a delayed invasion phase characterized by FN remodeling and LN re‐deposition that coincides with acinar proliferation and invasion. Mechanistically, it is shown that these changes are mediated by α3β1‐ and α5β1‐integrin–focal adhesion kinase (FAK) signaling, which in turn activates the mechanosensitive ion channel Piezo1 to regulate ECM composition, remodeling, and acinar invasion. Together, the findings reveal how mature epithelial structures dynamically respond to mechanical stiffening to create an invasive niche, offering new insights into how tissue architecture and stiffness synergize to drive breast cancer progression.

## Graphical abstract

Mechanical stiffening of the extracellular matrix around mature mammary acini induces sequential remodeling phases. An early response disrupts the basement membrane and promotes fibronectin secretion, followed by delayed invasion with matrix reorganization and proliferation. These events are coordinated by α3β1/α5β1‐integrin–FAK–Piezo1 signaling, uncovering a mechanosensitive pathway that links tissue architecture and stiffness to breast cancer progression.

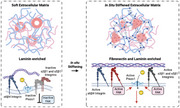

## 1 Introduction

The extracellular matrix (ECM), which comprises both, the basement membrane (BM) and the stromal ECM, plays a pivotal role in tissue morphogenesis and homeostasis, influencing cellular behaviors such as proliferation, differentiation and migration,^[^ ^1^, ^2^ ^]^ In the mammary gland, the mechanical properties of the ECM, such as its stiffness, are tightly regulated to drive morphogenesis and preserve tissue integrity.^[^ ^3^ ^]^ However, during tumorigenesis, the ECM undergoes extensive remodeling, leading to changes in its stiffness and composition and compromising its structural integrity,^[^ ^3^, ^4^ ^]^ These biomechanical changes drive malignant phenotypes in mammary epithelial cells (MECs) through loss of tissue polarity, disruption of the BM, increased proliferation and the acquisition of migratory phenotypes,^[^ ^5^, ^6^ ^]^ While considerable progress has been made in understanding how ECM stiffness promotes invasiveness in MECs and mammary acini isolated from their native ECM^[^ ^7^, ^8^, ^9^, ^10^ ^]^ the effects of increasing the stiffness around fully formed mammary acini within its self‐established ECM, which more accurately represents tumor progression, remains poorly understood.

Normal mammary acini, composed of a polarized epithelial monolayer surrounded by a BM, serve as a robust model for studying tissue‐specific responses to mechanical cues. The interplay between ECM mechanics, stromal ECM and BM remodeling, and cellular invasion is both dynamic and complex.^[^ ^6^ ^]^ For example, a stiff microenvironment can promote MECs to remodel BM through enhanced protease activity and generation of forces on the BM resulting in its disruption^[^ ^11^ ^]^ or through increased deposition of ECM proteins such as fibronectin (FN),^[^ ^12^, ^13^ ^]^ both leading to increased invasion and tumor progression. Similarly, changes in expression of BM modifying proteins such as netrin‐4 can lead to increases in its stiffness, facilitating cancer cell invasion and metastasis.^[^ ^14^ ^]^ Cancer cells can also utilize non‐proteolytic pathways to generate forces and squeeze through the pores of the ECM,^[^ ^11^, ^15^, ^16^ ^]^ However, the temporal dynamics and sequence of ECM remodeling events that occur when stiffness increases around fully formed mature epithelial structures, along with its consequence on proliferation and invasion are still not well characterized.

Mechanotransduction, the process by which cells sense and respond to mechanical stimuli, plays a central role in mediating cellular responses to ECM stiffening and integrins are key activators and transducers of cell‐ECM interactions in mammary acini.^[^ ^17^ ^]^ Integrins interact with both stromal ECM (FN, collagen) and BM (laminin (LN)) components to remodel ECM and regulate BM integrity through signaling pathways involving focal adhesion kinase (FAK) and others, several of which have been implicated in promoting tumor progression.^[^ ^18^, ^19^, ^20^, ^21^, ^22^, ^23^, ^24^ ^]^ In parallel, recent studies have also highlighted the role of mechanosensitive ion channels (MSCs), including Piezo1 and transient receptor potential (TRP) channels, in cellular responses to changes in ECM stiffness, implicating them in both normal physiology and cancer progression.^[^ ^25^, ^26^, ^27^, ^28^ ^]^ Intriguingly, emerging evidence suggests a potential interplay between integrin‐mediated signaling and MSCs in regulating stiffness‐induced tumor progression.^[^ ^29^, ^30^, ^31^, ^32^, ^33^ ^]^ However, how these pathways interact with each other in 3D tissues, both in the context of normal epithelial structures as well as in the context of mechanical ECM‐mediated changes in cancer tissues, still requires better understanding.

To answer these questions in physiological settings, here we utilized an in situ stiffening model of 3D mammary acini in their native ECM environment to simulate the increase in ECM stiffness observed during tumor progression. Using an interpenetrating network (IPN) hydrogel system composed of alginate and BM extract (BME), we grew fully mature mammary acini under soft (normal) conditions and induced stiffening through in situ crosslinking of the alginate once the acini were fully developed and had established their own ECM environment. This model allowed us to study how mechanical perturbations affect intact epithelial structures and how these structures, in turn, dynamically remodel their ECM and drive invasion.

Using this system, we identified a distinct program of ECM remodeling and invasion: an initial priming phase involving BM (LN) degradation and stromal ECM (FN) upregulation, followed by a delayed invasion phase characterized by FN remodeling and LN re‐secretion which coincided with the onset of proliferation and migration. Mechanistically, we find that that activation of Piezo1 downstream of β1 integrin‐FAK signaling is sufficient to trigger in situ stiffening‐induced invasion and ECM remodeling, thus establishing Piezo1 as an integrin effector protein. Together, these results provide new mechanistic insights into early mechanobiological transitions in tumorigenesis and the interplay between ECM remodeling, integrin signaling, and MSC activation.

## 2 Results

### 2.1 In Situ Stiffening of Normal Mammary Acini Results in Altered ECM Composition, Remodeling, and Distinct Invasive Phenotypes

**Figure 1:**
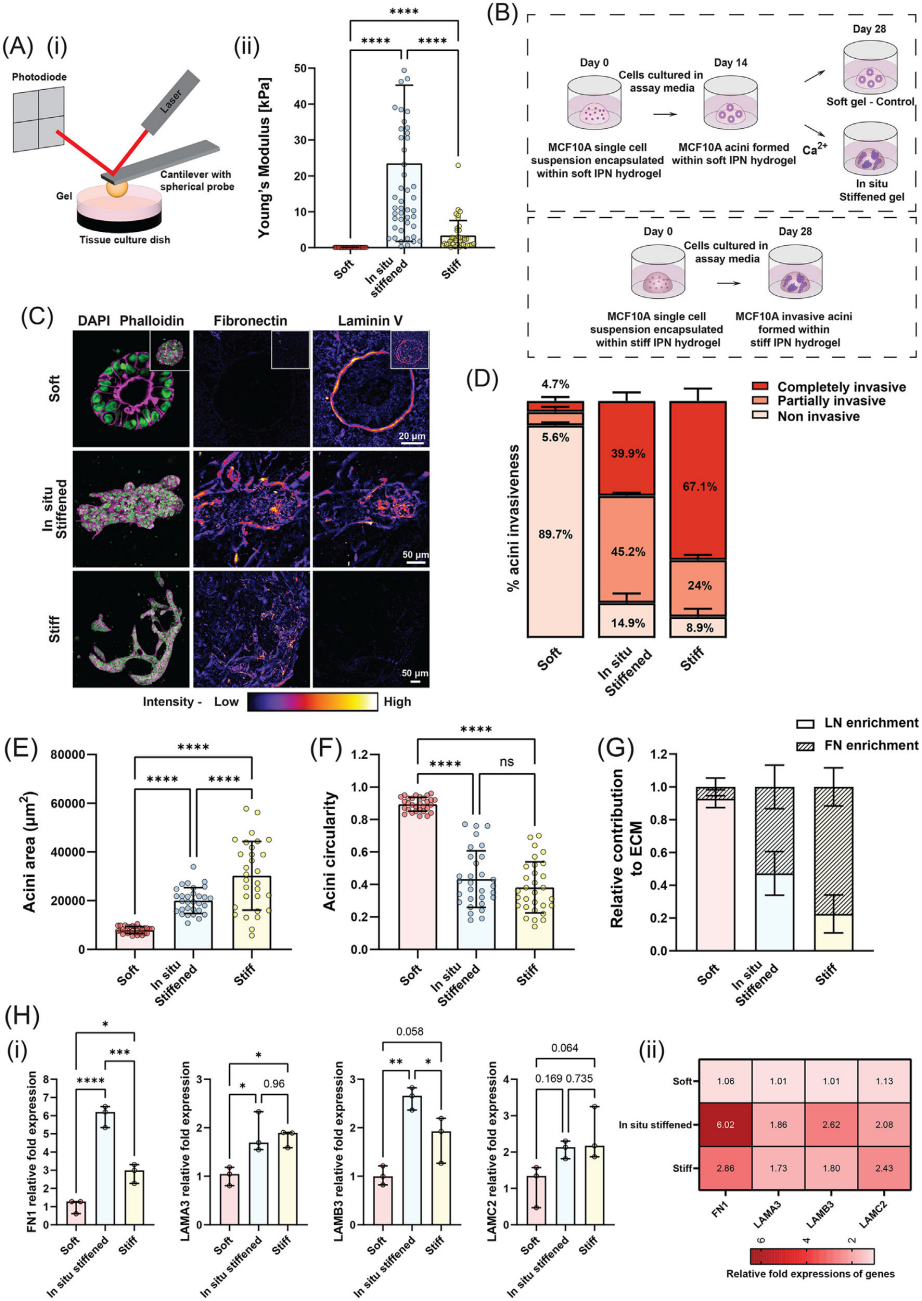
In situ stiffening of the normal mammary acini microenvironment induces distinct invasive phenotypes and dynamic ECM remodeling. A) Schematic representation of the AFM setup utilized for measuring the Young's modulus of soft, in situ stiffened, and stiff IPN gels. The AFM probe indents the gel surface to assess its mechanical properties across different stiffness conditions. AFM measurements of the Young's modulus of IPN gels under different stiffness conditions (n = 2 different experiments per condition, with 3 × 3 point measurements performed on three distinct regions in each experiment). Stiffness was measured immediately after the formation of soft and stiff gels and post‐ in situ stiffening of the soft gels. B) Schematic overview of the experimental workflow for 3D acini culture. Single‐cell suspensions of MCF10A were encapsulated in soft and stiff IPN gels. Once normal mature acini formed in soft IPN gels, a subset of the soft gels was in situ stiffened with 20 mM CaCl_2_ on day 14. Acini in unstiffened (soft control) and in situ stiffened IPN gels were cultured for an additional 14 days. Parallelly, acini within stiff IPN gels were cultured for 28 days. C) Confocal images showing nuclei (green), F‐actin (magenta), and FN and LN staining (fire LUT applied to highlight FN and LN levels within the acini) of acini in soft, stiffened and stiff IPN gels. Z‐stacked images of acini cultured in in situ stiffened and stiff IPN gels are shown, while a single Z‐plane (middle plane of the stack) image of acini cultured in soft IPN gels is displayed to highlight lumen formation (Z‐stacked image for the same acini is shown in the inset). Scale bars: 20 µm for soft; 50 µm for in situ stiffened and stiff. D–G) Quantification of (D) percentage of invasive acini, (E) acini area, (F) acini circularity, and (G) relative FN and LN enrichment in acini in soft, in situ stiffened, and stiff IPN gels (n = 30 acini from 3 different experiments, with 10 acini per experiment for acini area, circularity, and FN and LN enrichment quantifications; n ≥ 30 acini from 3 experiments for invasiveness quantification; Ordinary one‐way ANOVA test, ^****^ *p* < 0.0001, ns = non‐significant). H) (i) Quantification and (ii) heatmap of relative gene expression of FN1, LAMA3, LAMB3 and LAMC2 in acini embedded in soft, in situ stiffened and stiff IPN gels (n = 3 gels; Ordinary one‐way ANOVA test, ^*^ *p* < 0.05, ^**^ *p* < 0.01, ^***^ *p* < 0.001, ^****^ *p* < 0.0001). All changes are reported relative to β‐actin expression in acini grown in respective IPN gels.

To capture the process of stiffening‐induced changes in tissue organization, we adapted the IPN hydrogel system composed of alginate and BME to first grow 3D acini from single MECs in a mechanically soft environment^[^ ^9^ ^]^ and then cross‐linked the hydrogel in situ to stiffen it after formation of mature acini. We first confirmed that our system achieved the desired stiffness range through atomic force microscopy (AFM) measurements (**Figure** **1**A‐i). In situ crosslinking of the soft gel with 20 mM CaCl_2_, hereafter referred to as the in situ stiffened gel, increased stiffness from 86 ± 29 Pa to 24.8 ± 22.1 kPa (Figure 1A‐ii), effectively spanning the stiffness range of both normal and cancerous breast tissues.^[^ ^34^ ^]^ Additionally, we prepared stiff IPN gels by premixing 20 mM CaSO_4_^.^2H_2_O with the IPN gel mixture,^[^ ^9^ ^]^ which resulted in a stiffness of 3 ± 4.1 kPa (Figure 1A‐ii). Next, we encapsulated single‐cell suspensions of MCF10A MECs within the soft IPN gels and cultured them for 2 weeks to form fully mature mammary acini structures (Figure 1B). After the initial 2‐week culture period, a subset of gels was in situ stiffened by incubating them in 20 mM CaCl_2_ solution for 30 min. Following stiffening, the gels were washed three times with PBS to remove excess calcium and immediately returned to acini culture medium for continued growth. Acini in both unstiffened (soft) and in situ stiffened gels were then cultured for an additional 2 weeks (Figure 1B). All gels were subsequently fixed and stained for the nucleus (DAPI), F‐actin, LN (laminin‐332) and FN to measure acini morphology, BM integrity and stromal ECM deposition, respectively. In parallel, we also embedded single MECs in stiff gels and allowed them to grow for 28 days for comparison with the soft and in situ stiffened gels (Figure 1B).

Confocal microscopy of fixed acini revealed that most mature acini after 4 weeks of culturing in the soft IPN gels remained intact and spherical with fully developed lumens at their centers (Figure 1C; Figure S1, Supporting Information). In contrast, for in situ stiffened and stiff gels, we observed most acini to be significantly larger, disorganized and without an intact lumen though we did observe some variability in the levels of response (Figure 1C; Figure S1, Supporting Information). Notably, we found dramatic differences in ECM composition and remodeling between in situ stiffened and stiff gels. Normal mature acini in soft IPN gels had a clear and distinct layer of LN encircling each acinus with relatively faint or no FN staining around it (Figure 1C). However, while both in situ stiffened and stiff acini showed elevated secreted levels as well as fibrillogenesis of FN, there were dramatic differences in LN levels between the two conditions (Figure 1C). Invasive acini in in situ stiffened conditions showed elevated levels of LN staining with significantly remodeled LN that was in a discontinuous layer (Figure 1C). In contrast, in stiff gels, as previously reported,^[^ ^9^ ^]^ we found very low levels of LN along the invading fronts or anywhere else within the epithelial structure (Figure 1C). To confirm that calcium ions used for in situ crosslinking did not independently affect acinar morphology or ECM composition, we treated a subset of BME‐only gels (lacking alginate) containing mature acini with 20 mM CaCl_2_ for 30 min, replaced it with acini media and followed by continued culture for 2 weeks. This treatment had no observable effect on the stiffness of BME gels, as confirmed by AFM measurements (Figure S2A, Supporting Information), nor did it alter acinar architecture or FN and LN levels compared to acini in BME gels without calcium treatment, indicating that the phenotypic changes observed in in situ stiffened IPN gels are specifically attributable to matrix stiffening (Figure S2B, Supporting Information).To quantify the different acini structures observed, as well as capture the heterogeneity in acini response, we classified each acini as either non‐invasive (completely spherical, with an intact lumen), partially invasive (loss of spherical integrity to some extent, filling up of lumen/loss of lumen and invading cells sprouting out of the actual acini area) and completely invasive (complete loss of spherical integrity)(Figure 1F; Figures S1, S3, and S4A,B, Supporting Information). This analysis revealed that while normal mammary acini grown in soft IPN gels were ≈90% non‐invasive, stiffening of gels around fully formed normal acini resulted in ≈85% of the embedded acini to undergo morphological transformation with varied levels of invasion into the surrounding gel (Figure 1D–F; Figure S1, Supporting Information). Specifically, ≈40% lost their spherical structure, had filled‐in lumens and were completely invasive, while the rest exhibited partial invasiveness, characterized by protrusions sprouting from their surfaces while still maintaining their circular morphology and lumens (Figure 1D–F; Figure S1, Supporting Information). In contrast, growing single MECs in stiff IPN gels resulted in ≈90% of the acini to be invasive with a majority of these, ≈67%, being completely invasive with loss of lumen and no remaining spherical morphology (Figure 1D–F).

Within in situ stiffened gels, the deposition and remodeling of FN and LN varied according to the acini's response to the stiffening process (Figure S4C,D, Supporting Information). Non‐invasive acini exhibited robust LN signal encircling their periphery, indicative of an intact BM, and displayed minimal FN deposition. In partially invasive acini, FN levels were elevated, particularly at the invasive fronts, accompanied by a weak and discontinuous LN signal around the periphery, reflecting BM distortion (Figure S3, Supporting Information). Fully invasive acini, however, exhibited high levels of both FN and LN deposition, with these ECM components prominently localized at the periphery and within the acini (Figure S3, Supporting Information). To quantify these differences in ECM composition, we measured the overall background‐subtracted intensities of LN and FN staining and plotted the relative contribution of each component to the ECM in the acini i.e their relative levels within the ECM (Figure 1G; Figure S4C,D, Supporting Information). This analysis showed that while normal mammary acini were ≈80‐90% enriched with LN, in situ stiffening of the ECM around mature acini resulted in nearly equal contribution levels of the two (≈50%) which was mediated by partial reduction in LN deposition and significant increase in FN secretion (Figure 1G; Figure S4C,D, Supporting Information). In contrast, growing single MECs in stiff gels resulted in very low levels of LN leading to an ECM ≈80% enriched with FN (Figure 1G; Figure S4C,D, Supporting Information).

To further determine whether the changes in ECM deposition in mammary acini under different stiffness conditions were also associated with altered expression of ECM genes, we performed RT‐qPCR analysis of FN1 and LN‐332 subunits (LAMA3, LAMB3, LAMC2) across soft, in situ stiffened, and stiff gel conditions. FN1 expression was strongly upregulated after in situ stiffening compared to both soft and stiff gels. LAMA3 and LAMB3 expression were modestly but significantly increased following in situ stiffening, while LAMC2 expression remained unchanged (Figure 1H‐i, ii). These results suggest that the enhanced FN and LN deposition observed after in situ stiffening is at least partly transcriptionally regulated, although additional post‐transcriptional and matrix‐assembly mechanisms likely contribute.

To examine whether the observed response of normal MECs to in situ stiffening is also applicable to tumorigenic cell lines, we repeated the above experiments with tumorigenic MECs – MCF10DCIS.com and MCF7 cell lines. Consistent with previous reports, MCF10DCIS.com acini retained a generally circular morphology in soft IPN gels but failed to form lumens (Figure S5, Supporting Information).^[^ ^35^ ^]^ Similarly, MCF7 cells formed large, compact spheroid‐like structures lacking visible lumens in soft gels.^[^ ^36^ ^]^ Mature mammary structures formed in soft IPN gels by both cell lines exhibited multiple breaches in the basement membrane with MCF7 spheroids showed markedly higher FN deposition compared to MCF10DCIS.com structures, which exhibited minimal FN enrichment. Upon in situ stiffening, both cell types underwent pronounced morphological changes and adopted invasive phenotypes. MCF10DCIS.com acini formed protrusive structures and invaded into the surrounding matrix, whereas MCF7 spheroids became disorganized and exhibited collective invasion (Figure S5, Supporting Information). This transition to an invasive state was accompanied by extensive ECM remodeling, characterized by robust FN accumulation and a modest increase in LN levels around the invading structures. These findings suggest that in situ stiffening alone is sufficient to trigger invasive behavior and ECM remodeling in both preinvasive and luminal‐like breast cancer models.

Together, these results demonstrate that in situ stiffening of normal mammary acini within their native BM‐ECM induces invasive phenotypes via dynamic remodeling of that ECM, a process that is distinct from those of acini grown in pre‐stiffened environments.

### 2.2 In Situ ECM Stiffening Results in Distinct Phases of Changes in ECM Composition, Remodeling, and Onset of Invasion in Mammary Acini

**Figure 2:**
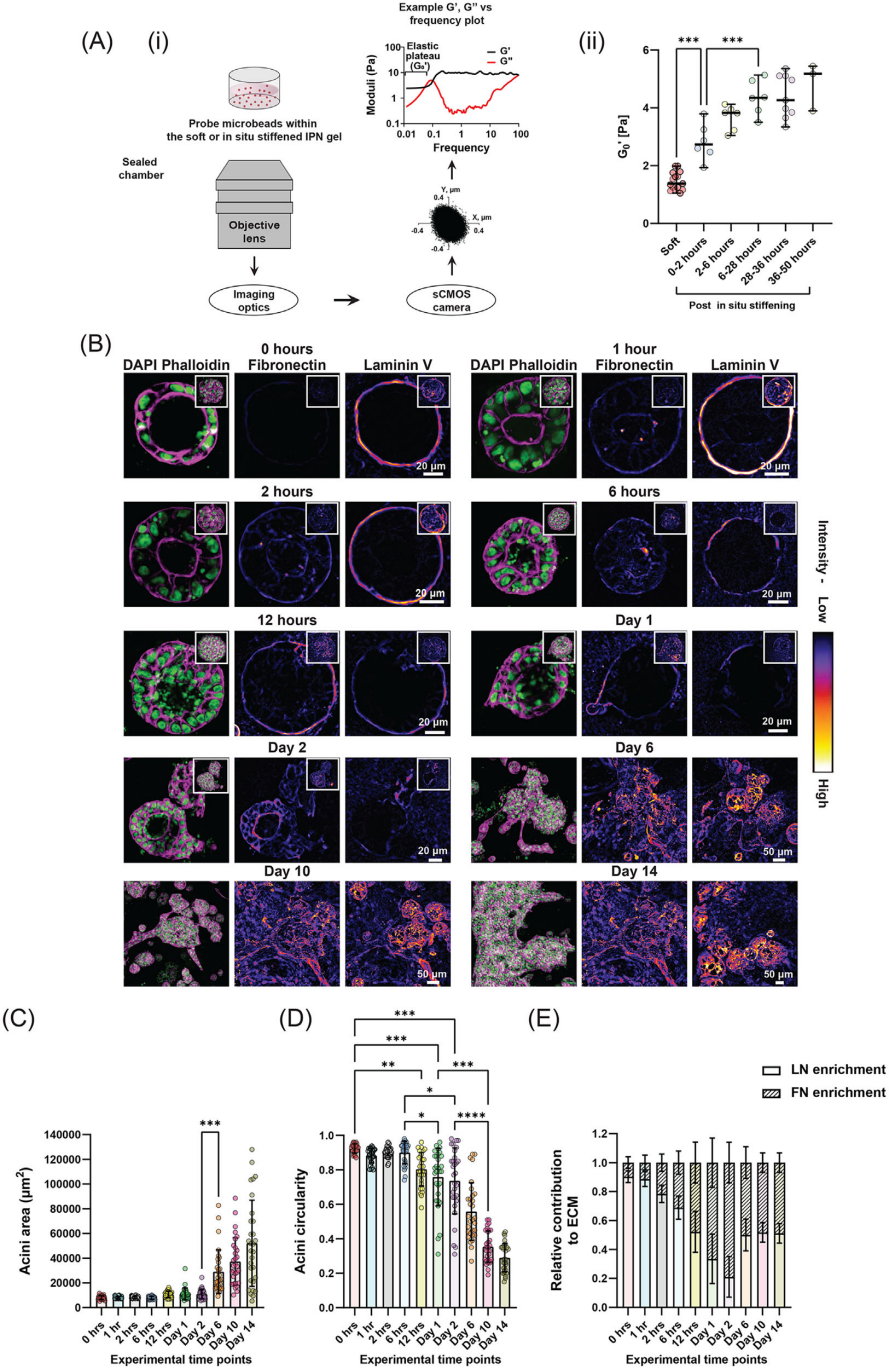
In situ ECM stiffening results in distinct phases of changes in ECM composition, remodeling and onset of invasion in mammary acini. A) (i) Schematic representation of the imaging setup used for PTM to measure the local stiffness of soft, IPN gels before and after the in situ stiffening process. The schematic includes an example of a small region of interest image of the probe, where the Brownian motion of the probe is tracked using a centre‐of‐mass algorithm to extract the low‐frequency elastic modulus (G'_0_) of the material surrounding the probe. (ii) Low‐frequency elastic modulus (G'_0_) of the soft IPN gel measured via PTM before and after the in situ stiffening process (n = 2 gels per condition, with 3 beads in different regions of each gel; Ordinary one‐way ANOVA test, ^***^ *p* < 0.001). Stiffness measurements for the soft IPN gels were recorded over the first 24 h post‐gelation. The same gels were subsequently in situ stiffened, and measurements were taken over the following 2 days to monitor changes in stiffness post‐modulation. B) Confocal images of nuclei (green), F‐actin (magenta), and FN and LN (fire LUT applied to highlight FN and LN levels within the acini) staining at various time points post‐in situ stiffening of soft IPN gel. Z‐stacked images of acini within in situ stiffened IPN gels are shown for Days 6, 10, and 14 post‐stiffening. Single Z‐plane images (middle plane of the stack) are displayed for time points 0 h, 1 h, 2 h, 6 h, 12 h, Days 1, and 2 post‐stiffening to illustrate lumen filling and early invasion events (Z‐stacked images for the same acini are shown in the insets). Scale bars: 20 µm for time points – 0 h, 1 h, 2 h, 6 h, 12 h, Days 1 and 2, 50 µm for time points – Days 6, 10, and 14, 50 µm. C–E) Quantification of (C) acini area, (D) acini circularity, and (E) relative FN and LN enrichment in acini at various time points post‐in situ stiffening of the soft IPN gel (n = 30 acini from 3 different experiments, with 10 acini per experiment for acini area, circularity, and FN and LN enrichment quantifications; n ≥ 30 acini from 3 experiments for invasiveness quantification; Kruskal‐Wallis test, ^*^ *p* < 0.05, ^**^ *p* < 0.01, ^***^ *p* < 0.001, ^****^ *p* < 0.0001).

Having established a system that allows for in situ stiffening of fully mature normal acini in their native ECM environment, we next aimed to investigate the temporal progression of morphological and ECM remodeling events during this process. To do so, we employed particle tracking microrheology (PTM) in addition to AFM to map the dynamics and spatial heterogeneity of the in situ stiffening process,^[^ ^37^, ^38^ ^]^ While AFM provides absolute values of local gel stiffness at the surface of the gel, PTM enabled us to follow the temporal evolution of viscoelastic changes and local variability within the bulk of the gel. Briefly, 6 mm diameter polystyrene beads were embedded in soft IPN gels, incubated for 24 h, and then imaged on a widefield microscope with a high acquisition rate, ≈300 Hz, for several minutes to observe the Brownian motion and diffusion of the beads. After collecting data on the soft gels over 22 h (measurements taken every 2 h for 10 h per day), the gels were crosslinked using 20 mM CaCl_2_ to induce stiffening and the embedded beads were further tracked over 2 days. For each set of tracked data, the mean square displacement (MSD) of the bead over time was determined and used to measure the low‐frequency plateau of the elastic modulus G’_0_ (**Figure** **2**A‐i, see Experimental Section). Plotting G’_0_ over time revealed that the gels underwent a rapid increase in stiffness within the first 6 h (≈3.5‐fold increase compared to the soft gels), followed by a continued stiffening up to 24 h post‐crosslinking, after which the stiffness plateaued and remained stable for the subsequent 24 h (Figure 2A‐ii). These measurements show that in situ stiffening occurs quickly, with most changes in mechanical properties complete within a day and then remains stable over longer timescales.

Next, we embedded single MCF10A MECs in soft IPN gels and allowed them to form normal mature acini for 14 days, just as above. After in situ crosslinking with 20mM CaCl_2_, subsets of in situ stiffened gels were fixed at 6 h, 12 h, 24 h, 2 days, 6 days, 10 days, and 14 days post stiffening and stained for DAPI, F‐actin, FN, and LN. Confocal imaging and quantification of acini morphology and LN and FN levels under these conditions revealed two temporally distinct phases of changes induced by in situ stiffening, a rapid ECM response followed by delayed morphological changes and invasion (Figure 2B–E; Figure S6A–D, Supporting Information). At 6 h post‐stiffening, we observed a robust increase in FN levels around the periphery of the acini with concurrent reduction in LN levels, which reduced the enrichment of LN levels by twofold (Figure 2B,E; Figure S6A–D, Supporting Information). The acini however remained intact and circular during this initial 6‐h period with no increase in area or loss of circularity (Figure 2C,D). At 12 h, some acini began to show luminal infiltration and while there was some indication of an increase in area and loss in circularity, this was not significant, however the LN levels continued to significantly reduce, and FN levels continued to increase in the surrounding ECM (Figure 2B–E; Figure S6A–D, Supporting Information). This process continued in samples fixed 24 and 48 h post‐stiffening, and by 48 h, we observed cells beginning to invade into their surrounding environment with high FN signal localized around and through the invading front and no LN signal at these regions (Figure 2B–E; Figure S6A–D, Supporting Information). Quantification revealed only a small change in acini area and circularity at 48 h with most of the LN gone from around the acini resulting in a FN‐enriched ECM (with an enrichment index of ≈0.8) (Figure 2B–E; Figure S6A–D, Supporting Information).

This priming phase was followed by a second phase observed in samples fixed 6 days post‐stiffening, where LN levels increased significantly compared to 2 days resulting in the acini being equally enriched for LN and FN levels (Figure 2B; Figure S6A–D, Supporting Information). This enrichment was driven by re‐secretion of LN as FN levels continued to remain high (Figure S6A–D, Supporting Information). The acini now lost most of their circular morphology and invaded significantly into their surrounding with high FN and LN levels at the invading fronts (Figure 2B–E; Figure S6A–D, Supporting Information). The acini continued to invade and expand, were no longer circular and high levels of both, FN and LN were maintained during subsequent timepoints until day 14 (Figure 2B–E; Figure S6A–D, Supporting Information).

Taken together, these results reveal a temporally distinct two‐phase response to in situ stiffening: a rapid ECM priming phase characterized by FN deposition and BM disruption, followed by a delayed invasion phase driven by LN re‐secretion and ECM reorganization.

### 2.3 β1 Integrin–FAK Signaling Regulates ECM Remodeling and Invasion in Response to In Situ Stiffening

Mechanical signals from the ECM are transduced into biochemical responses primarily through integrins and their downstream effectors, such as FAK, which play critical roles in cell‐matrix interactions and mechanotransduction. Prior studies have highlighted the role of integrin‐mediated signaling in driving invasive phenotypes in MECs^[^ ^19^, ^21^, ^39^ ^]^ Given our observations of pronounced changes in the ECM, marked by initial LN loss and its subsequent increased deposition and FN fibrillogenesis in in situ stiffened acini, we hypothesized that integrin‐FAK signaling might mediate stiffening‐induced morphological changes and invasive transitions. To test this, we investigated the expression and activation of β4 integrins,^[^ ^40^ ^]^ which primarily bind LN, and β1 integrins which interact with FN,^[^ ^39^ ^]^ in acini grown in soft, in situ stiffened, and stiff IPN gels.

**Figure 3:**
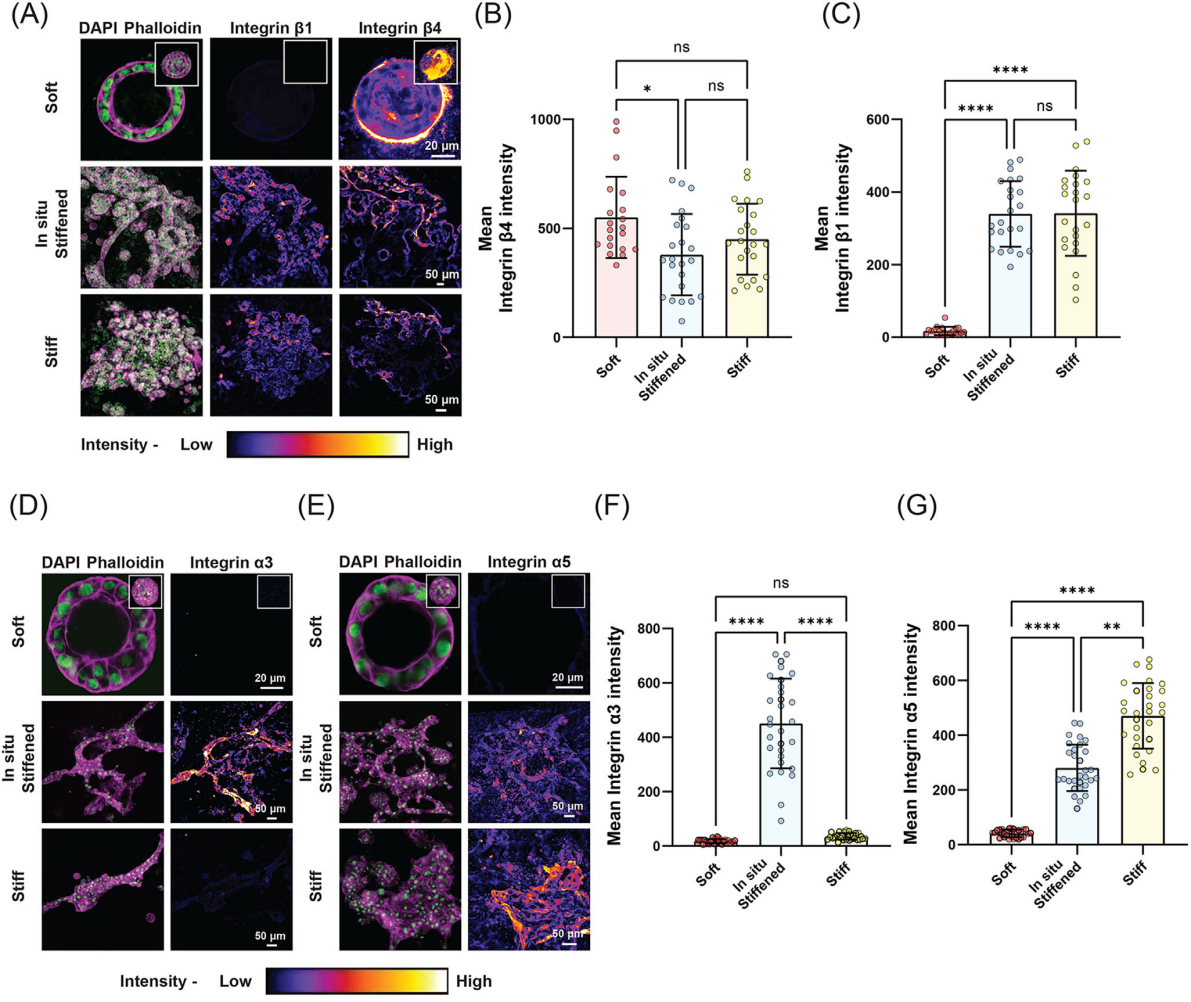
Integrins drive ECM remodeling in acini. A) Confocal images of acini cultured in soft, in situ stiffened, and stiff IPN gels, showing nuclei (green), F‐actin (magenta), β1 integrin, and β4 integrin (fire LUT applied to highlight β1 and β4 integrin expression). Z‐stacked images are displayed for in situ stiffened and stiff gels, while a single Z‐plane image of acini in soft gels highlights lumen formation (Z‐stacked image for the same acini in inset). Scale bars: soft, 20 µm; in situ stiffened and stiff, 50 µm. B,C) Quantification of mean (B) β1 integrin and (C) β4 integrin intensities within acini cultured in soft, in situ stiffened, and stiff IPN gels (n = 20 acini from 2 different experiments, with 10 acini per experiment; Kruskal‐Wallis test, ^*^ *p* < 0.05, ^****^ *p* < 0.0001, ns = not significant). D,E) Confocal images of acini cultured in soft, in situ stiffened, and stiff IPN gels, showing nuclei (green), F‐actin (magenta), α3 integrin, and α5 integrin (fire LUT applied to highlight α3 and α5 integrin expression). Z‐stacked images are displayed for in situ stiffened and stiff gels, while a single Z‐plane image of acini in soft gels highlights lumen formation (Z‐stacked image for the same acini in inset). Scale bars: soft, 20 µm; in situ stiffened and stiff, 50 µm. F,G) Quantification of mean (B) α3 integrin and (C) α5 integrin intensities within acini cultured in soft, in situ stiffened, and stiff IPN gels (n = 20 acini from 2 different experiments, with 10 acini per experiment; Kruskal‐Wallis test, ^*^ *p* < 0.05, ^****^ *p* < 0.0001, ns = not significant).

To do so, we first stained acini grown in soft, in situ stiffened and stiff IPN gels for total β4 integrins and active β1 integrins that bind to FN. Confocal imaging of normal acini in soft conditions showed a distinct circular and continuous localization of β4 integrins surrounding the intact acini that resembled LN staining in Figure 1 (**Figure** **3**A). Even though β1 integrins can dimerize with α6 and α3 integrins to bind to LN,^[^ ^41^ ^]^ staining for the β1 subunit in soft gels showed a faint and comparatively lower signal around the acini as well as everywhere else in proximity of the epithelial structure (Figure 3A). This was confirmed through quantification of intensity and levels of active β1 integrins (relative to total β4) which showed overall low levels of β1 activation (Figure 3B,C; Figure S7A–C, Supporting Information). In comparison to the acini in soft gels, we found unexpected changes in integrin levels in acini within stiff and in situ stiffened gels (Figure 3A). First, both in situ stiffened and stiff acini with invasive phenotypes continued to exhibit elevated levels of total β4 integrins (Figure 3A), which contrasted with the observed differences in LN levels between acini in stiff and in situ stiffened gels. In addition, not surprisingly, high levels of active β1 integrin staining were now observed throughout the invasive acinar regions correlating with the FN localization pattern in Figure 1 (Figure 3A). This led to an overall significant increase in enrichment of active β1 integrins at the acini‐ECM interface in in situ stiffened and stiff conditions compared to acini grown in soft gels (Figure 3B,C).

To further investigate the integrin repertoire involved in stiffening‐induced transitions, we also stained for α3 and α5 integrins, which are known dimerization partners of β1 integrins. In acini within soft gels, α3 and α5 integrin levels were very low or undetectable (Figure 3D,E). Upon in situ stiffening, α3 integrin expression increased significantly and was prominently localized at the acini–ECM interface, whereas stiff gels exhibited variable but overall reduced α3 signal (Figure 3D). In contrast, α5 integrin staining was markedly upregulated in both in situ stiffened and stiff gels, with strong peripheral localization corresponding to regions of FN deposition (Figure 3E). Quantification confirmed that both α3 and α5 integrins showed significant increases in intensity in in situ stiffened conditions compared to soft gels, with α5 integrins particularly enriched in regions of FN fibrillogenesis (Figure 3F,G; Figure S7D,E). Taken together, these results indicate that in situ stiffening results in activation of α3β1 and α5β1integrins that drives acini proliferation and invasion. This mechanism is distinct from acini invasion under stiff conditions which is primarily dominated by α5β1.

**Figure 4:**
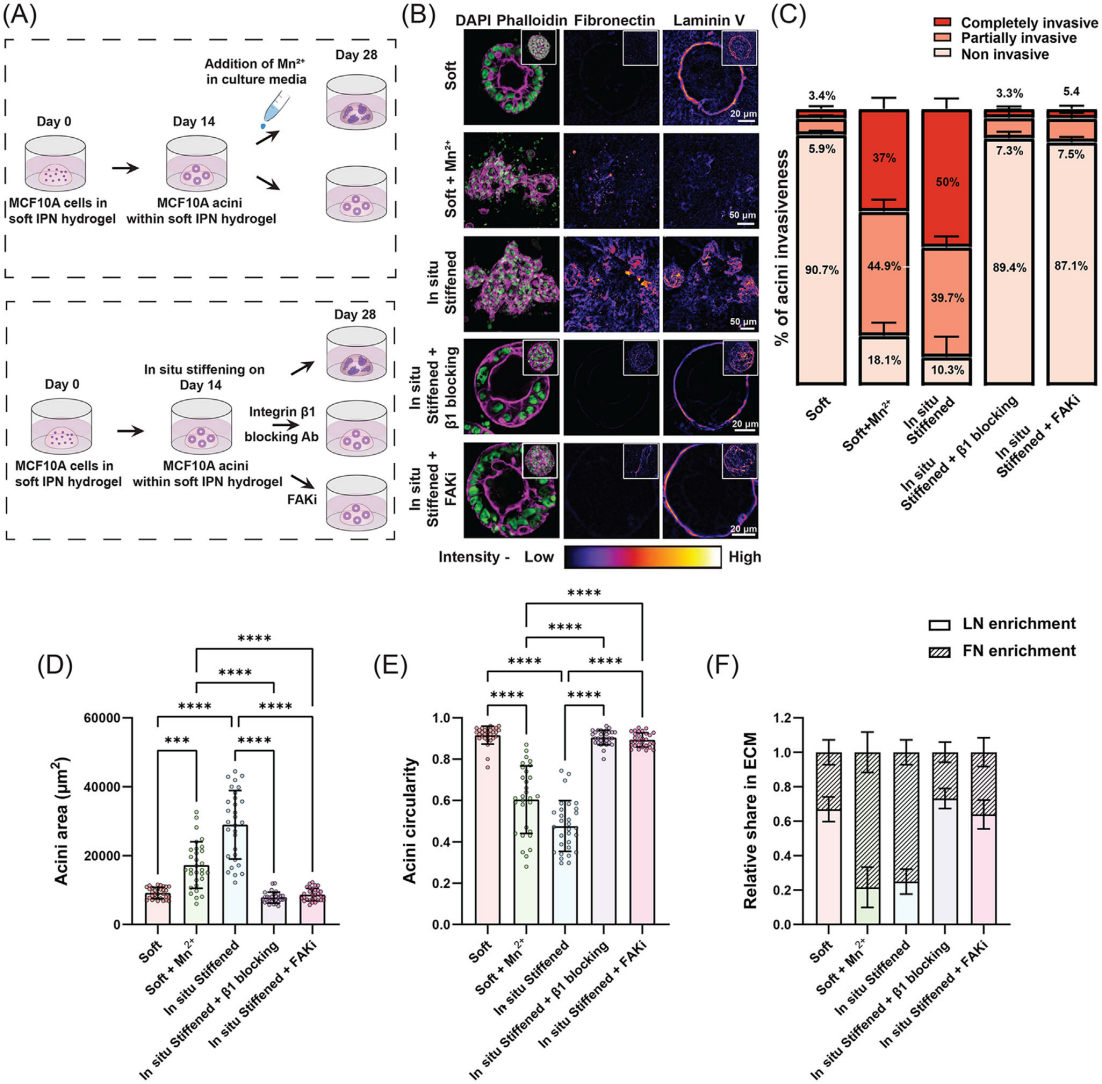
β1 Integrin–FAK axis drives ECM remodeling and in situ stiffening‐induced invasion. A) Schematic representation of the experimental workflow to investigate integrin‐dependent phenotypic alterations in acini. After mature acini formation in soft IPN gels, gels were: (i) left untreated (soft control), (ii) treated with MnCl_2_ to activate integrins, (iii) in situ stiffened and either left untreated (in situ stiffened control), or treated with integrin β1 blocking antibody or FAKi. B) Confocal images showing nuclei (green), F‐actin (magenta), and FN and LN (fire LUT applied) in acini cultured under various experimental conditions. Z‐stacked images are shown for acini in soft + Mn^2^⁺ and in situ stiffened gels, while single Z‐plane images highlight lumen formation and retention in soft, in situ stiffened + β1 blocking, and in situ stiffened + FAKi conditions (Z‐stacked image for the same acini in inset). Scale bars: 20 µm for soft, in situ stiffened + β1 blocking, and in situ stiffened + FAKi; 50 µm for soft + Mn^2^⁺ and in situ stiffened. C–F) Quantification of (C) percentage of acini invasiveness, (D) acini area, (E) acini circularity, and (F) relative FN and LN enrichment in different experimental conditions (n = 30 acini from 3 different experiments, with 10 acini per experiment for area and circularity quantifications; n ≥ 30 acini from 3 experiments for invasiveness; Kruskal‐Wallis test, ^***^ *p* < 0.001, ^****^ *p* < 0.0001).

Since our data above indicated a role for activation of integrins, we next tested if activation was sufficient to result in invasive phenotypes in normal mammary acini. To test this, we treated fully mature normal acini grown in soft gels with 0.5mM MnCl_2_ that promotes the activation of integrins independent of stiffness (**Figure** **4**A).^[^ ^42^ ^]^ Upon MnCl_2_ treatment, we found that ≈88% of the normal mature acini in soft gels now exhibited an invasive phenotype as quantified by significant loss of acini circularity and increase in area (Figure 4B–E; Figure S8A,B, Supporting Information). However, compared to acini in in situ stiffened gels, MnCl_2_ treated acini expanded less and did not produce FN to the same level (Figure 4F; Figure S8C, Supporting Information). Furthermore, we found that LN levels in these acini were also significantly lower compared to acini in in situ stiffened gels (Figure 4F; Figure S8D, Supporting Information). This suggests that while integrin activation is sufficient to promote invasion of mammary acini in the soft environment, in situ stiffening activates other pathways or amplifies integrin pathways to fully recapitulate the invasive phenotype.

We next investigated the specific role of β1 integrins in this process by inhibiting the activity of β1 integrins using the specific inhibitory antibody AIIB2 or its downstream target FAK during in situ stiffening of normal mature acini (Figure 4A). After two weeks of culturing in the in situ stiffened gel, mammary acini in the presence of the integrin activation blocking antibody or the FAK inhibitor did not undergo any invasive transformation, maintaining their confined area, circularity, lumen and FN and LN levels similar to those in the soft control group (Figure 4B–F; Figure S8A–D, Supporting Information). Quantification showed that over 90% of the in situ stiffened acini treated with integrin β1‐blocking antibody or the FAK inhibitor remained non‐invasive, i.e., showed ≈ninefold reduction compared to the in situ‐stiffened control group, thus rescuing the phenotype completely (Figure 4C).

Taken together, these results show that in situ stiffening of normal mature mammary acini results in specific increase in activation of β1 integrins and signaling via FAK which drives ECM remodeling and invasive transitions.

### 2.4 Mechanosensitive Ion Channels Act Downstream of Integrins to Mediate In Situ Stiffening‐Induced Invasion

Multiple studies have demonstrated that MSCs such as TRP ion channels and Piezo1 are associated with ECM stiffness‐driven cancer progression,^[^ ^25^, ^26^ ^]^ Recent work also suggests significant crosstalk between MSCs and integrin‐mediated focal adhesion signaling, that transduce ECM stiffness cues in various cell types,^[^ ^27^, ^28^, ^43^ ^]^ As we found that integrin activation did not, on its own completely phenocopy the in situ stiffened phenotype, we hypothesized that activation of MSCs might function as another effector in this process.

**Figure 5:**
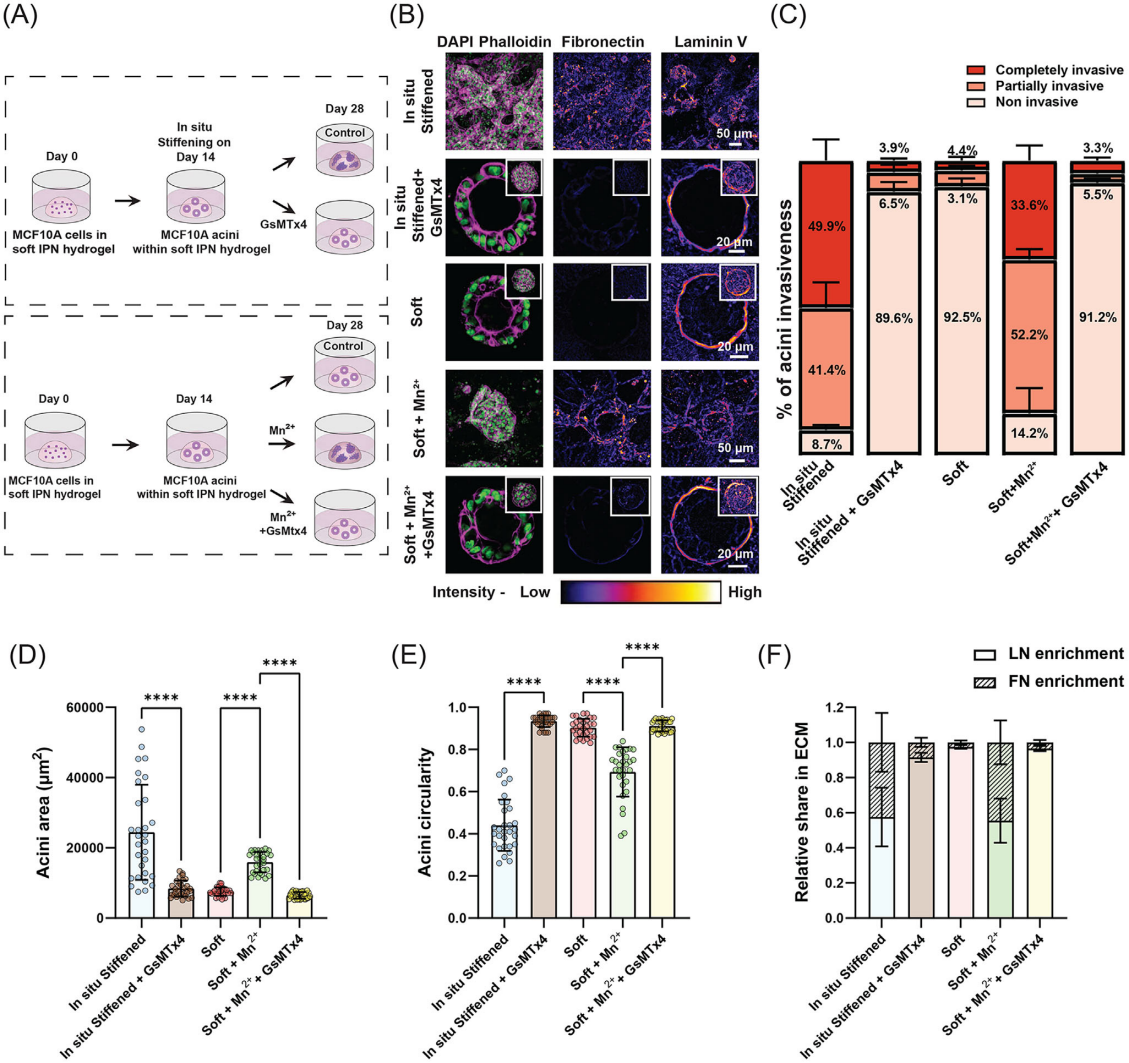
MSCs mediate in situ stiffening induced invasion downstream of integrin activation. A) Schematic overview of the experimental overflow to test if MSCs are associated and crosstalk with integrins during the in situ stiffening mediated invasion in normal mammary acini. Once the mature acini have formed in the soft IPN gels, some gels – (i) were in situ stiffened which were then either cultured to serve as in situ stiffened control or were incubated with media supplemented with GsMTx4, a MSCs inhibitor, (ii) served as soft control, (iii) were incubated with media supplemented with MnCl_2_ (±) GsMTx4 to simultaneously block the activity of MSCs and activate integrins. B) Confocal images showing nuclei (green), F‐actin (magenta), and FN and LN (fire LUT applied) in acini cultured under various experimental conditions. Z‐stacked images are shown for acini in soft + Mn^2^⁺ and in situ stiffened gels, while single Z‐plane (middle plane image of the Z stack) images highlight lumen formation and retention in soft, soft + Mn^2^⁺ +GsMTx4, and in situ stiffened + GsMTx4 conditions (Z‐stacked images for the same acini in inset). Scale bars: 20 µm for soft, soft + Mn^2^⁺ +GsMTx4, and in situ stiffened + GsMTx4; 50 µm for soft + Mn^2^⁺ and in situ stiffened gel samples. C–F) Quantification of (C) percentage of acini invasiveness, (D) acini area, (E) acini circularity, and (F) relative FN and LN enrichment in different experimental conditions (n = 30 acini from 3 different experiments, with 10 acini per experiment for area and circularity quantifications; n ≥ 30 acini from 3 experiments for invasiveness quantification; Kruskal‐Wallis test, ^****^ *p* < 0.0001).

To test this, we inhibited the activity of MSCs using GsMTx4, a spider venom peptide that inhibits several MSCs including Piezo1 and TRP channels, in normal mammary acini during the stiffening process (**Figure** **5**A). Single MECs were embedded in soft IPN gels, cultured to form mature acini over two weeks, and then in situ stiffened with or without GsMTx4 for an additional two weeks. Confocal microscopy revealed that GsMTx4‐treated acini maintained their circular morphology and hollow lumens, in contrast to the invasive phenotypes observed in untreated in situ stiffened acini (Figure 5B–E; Figure S9A,B, Supporting Information). Furthermore, GsMTx4 treatment reduced FN and increased LN levels, restoring both their total and relative levels to those observed in normal acini in soft gels (Figure 5F; Figure S9C,D, Supporting Information). Quantification confirmed that MSC inhibition significantly decreased the proportion of invasive acini, reducing the fraction of partially and completely invasive acini from ≈90% in untreated in situ stiffened gels to ≈10% in GsMTx4‐treated gels (Figure 5C). This was accompanied by a restoration of acini circularity and area to levels comparable to those in soft gels (Figure 5D,E; Figure S9A,B, Supporting Information).

To determine whether MSC activation was upstream or downstream of integrin and FAK activation, we then treated fully mature acini in soft gels with both MnCl_2_, which activates integrins independently of stiffness, and GsMTx4 (Figure 4A). Following the dual treatment, mammary acini showed no invasion maintaining confined area, circularity, lumen structure and relative levels of FN and LN, similar to normal mammary acini grown in soft gels (Figure 5B–F; Figure S9A–D, Supporting Information). This was in stark contrast to acini cultured in soft gels treated only with MnCl_2_ which exhibited the invasive phenotype and different LN and FN levels (Figure 5B–F; Figure S9A–D, Supporting Information). Quantification confirmed that while activating integrins with MnCl_2_ increased the number of partially invasive and completely invasive acini from ≈10% in soft gels to ≈85%, this was completely rescued by addition of GsMTx4 to ≈8% of invasive acini (Figure 5C). This rescue of invasive phenotype was through the same pathway of ECM regulation as addition of GsMTx4 to MnCl_2_ treated acini in soft gels significantly increased peripheral levels of LN and reduced FN deposition (Figure 5B,F). Taken together, these results demonstrate that MSC activation is a necessary downstream effector of integrin signaling that results in stiffening‐induced acinar invasion.

### 2.5 Piezo1 Mediates β1 Integrin–FAK Dependent ECM Remodeling and Invasion in Response to In Situ Stiffening

Our findings discussed above demonstrate that in situ stiffening induces the invasive phenotype in normal mammary acini through MSC activation downstream of integrin signaling. To identify the specific MSC mediating this process, we focused on Piezo1, a critical MSC known to interact with β1 integrin signaling pathways and regulate mechanotransduction,^[^ ^27^, ^29^, ^31^, ^33^, ^44^ ^]^ Piezo1 has been implicated in ECM‐driven cellular processes such as migration and invasion,^[^ ^29^, ^32^, ^33^, ^45^, ^46^ ^]^ making it a strong candidate for mediating stiffening‐induced invasion in 3D mammary acini.

**Figure 6:**
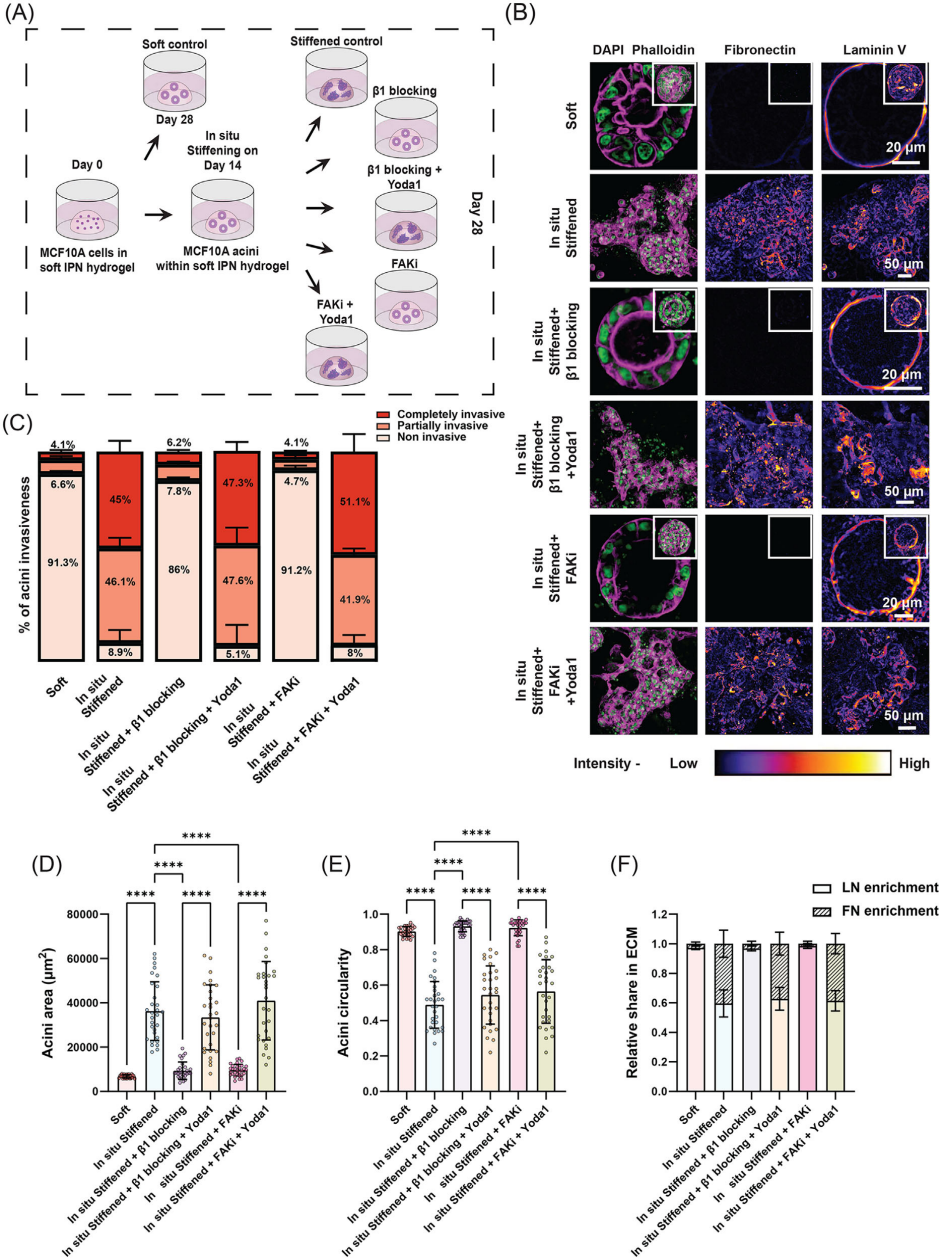
Piezo1 drives invasion downstream of β1 Integrin–FAK signaling to regulate in situ stiffening mediated changes in ECM composition and remodeling. A) Schematic overview of the experimental workflow to examine if Piezo1 MSC acts downstream to β1 Integrin–FAK Axis during the in situ stiffening mediated mechanotransduction in normal mammary acini. After mature acini formation in soft IPN gels, gels were: (i) left untreated (soft control), (ii) in situ stiffened and either left untreated (in situ stiffened control), or treated with integrin β1 blocking antibody or FAKi (±) Yoda1, a Piezo1agonist. B) Confocal images showing nuclei (green), F‐actin (magenta), and FN and LN (fire LUT applied) in acini cultured under various experimental conditions. Z‐stacked images are shown for acini in in situ stiffened, in situ stiffened + β1 blocking + Yoda1, and in situ stiffened + FAKi + Yoda1 conditions, while single Z‐plane images are shown to highlight lumen formation and retention in soft, in situ stiffened + β1 blocking, and in situ stiffened + FAKi conditions (Z‐stacked image for the same acini in inset). Scale bars: 20 µm for soft, in situ stiffened + β1 blocking, and in situ stiffened + FAKi; 50 µm for in situ stiffened, in situ stiffened + β1 blocking + Yoda1, and in situ stiffened + FAKi + Yoda. C–F) Quantification of (C) percentage of acini invasiveness, (D) acini area, (E) acini circularity, and (F) relative FN and LN enrichment in different experimental conditions (n = 30 acini from 3 different experiments, with 10 acini per experiment for area, circularity and FN and LN enrichment quantifications; n ≥ 30 acini from 3 experiments for invasiveness quantification; Kruskal‐Wallis test, ^****^ *p* < 0.0001).

To investigate the role of Piezo1, we embedded single MECs in soft IPN gels and cultured them for 14 days to form normal mammary acini. We then in situ stiffened the gels under five different conditions: 1) standard in situ stiffened conditions for 14 days, 2) in situ stiffened with β1 integrin blocking antibody (AIIB2), 3) in situ stiffened with FAK inhibitor, 4) in situ stiffened with β1 integrin blocking antibody plus the Piezo1 agonist Yoda1, and 5) in situ stiffened with FAK inhibitor plus Yoda1 (**Figure** **6**A). All samples were fixed, stained for LN and FN, and analyzed alongside normal mammary acini grown in soft gels for 28 days. In addition to the pharmacological experiments, we performed RT‐qPCR analysis to assess Piezo1 expression under soft, in situ stiffened, and stiff conditions. This revealed that Piezo1 transcript levels were significantly elevated in acini from in situ stiffened and stiff gels compared to soft gels (Figure S10A,B, Supporting Information), suggesting that in situ stiffening and stiff ECM promotes the expression of Piezo1 and thus supporting its role in mediating the observed invasive phenotypes.

Confocal microscopy revealed that as shown above, in situ stiffening of normal mammary acini resulted in invasive phenotypes which were rescued by blocking activation of β1 integrin or inhibition of FAK (Figure 6B). However, we observed that this blocking effect was lost in the presence of Yoda1, both in terms of morphology of the acini as well as levels of LN and FN along the invading fronts in the ECM (Figure 6B). Quantification of acini invasiveness showed that while inhibiting β1 integrin‐FAK signaling reduced partially and completely invasive acini by six and sevenfold, this effect was lost in the presence of Yoda1 which resulted in complete restoration of invasive acini to levels seen in in situ stiffened conditions as well as in overall acini size and circularity (Figure 6C–E; Figure S11A–D, Supporting Information). Additionally, quantification of LN and FN showed that activation of Piezo1 in in situ stiffened acini in the presence of β1‐FAK inhibition was sufficient to down‐regulate LN levels and upregulate FN levels in the ECM which correlated with the increased invasiveness observed under these conditions (Figure 6F; Figure S11A–D, Supporting Information). Taken together, these results show that Piezo1 is a key MSC that acts downstream of in situ stiffening mediated β1 integrin and FAK signaling and regulates ECM remodeling to drive invasion in mammary acini.

## 3 Discussion

Here, we show that in situ stiffening of fully developed mammary acini triggers a dynamic and multi‐phase invasion process, driven not only by mechanical cues sensed by the cells but also by their feedback on the surrounding ECM. This process begins with stiffening‐induced priming of the acinar environment, characterized by downregulation or degradation of the BM and increased stromal ECM production around the acini structure. This is followed by a second phase marked by increased cellular proliferation and migration that coincides with upregulated BM production and further stromal ECM remodeling. Mechanistically, we find that in situ stiffening–induced ECM remodeling and invasion is triggered by increased activation of FN‐binding β1 integrins and its downstream kinase FAK. Critically, we identify that the mechanosensitive ion channel Piezo1 is the downstream effector of this process, activation of which in the presence of the stiffened ECM is necessary and sufficient to drive invasion.

The invasive behaviors seen in in situ stiffened acini are phenotypically and mechanistically distinct from those observed when single MECs are grown in pre‐stiffened matrices (stiff IPN gels). In stiff gels, cells experience high rigidity continuously from the point of encapsulation, which disrupts α6β4 integrin–LN clustering and prevents proper hemidesmosome formation, thereby abolishing apicobasal polarity and basement membrane deposition.^[^ ^9^ ^]^ Consequently, acini in stiff gels fail to form organized structures, exhibit reduced β4 integrin and LN, and display partial, scattered invasion. By contrast, acini in in situ stiffened gels first develop in a compliant environment that allows normal morphogenesis and basement membrane assembly before encountering an increase in rigidity. This dynamic change produces a distinct invasion phenotype, characterized by FN deposition, LN remodeling, and collective invasion from an organized, polarized epithelial structure. These findings emphasize that dynamic mechanical changes over time are not equivalent to static stiffness exposure: mechanical history and timing are crucial determinants of the invasion program. While final stiffness values may further influence the magnitude of integrin signaling, contractility, and invasion, it is the temporal sequence of stiffening superimposed on a pre‐formed epithelial architecture that makes the in situ system mechanistically and phenotypically distinct.

By following the invasion process over time, we identify two key features. First, the rapid ECM remodeling immediately after stiffening—defined by FN upregulation and LN depletion—marks a “priming” phase preceding morphological change. We propose that this FN surge is a key initial response to matrix stiffness and speculate it may arise from either BM‐dependent mechanotransduction (e.g., α6β4 integrins) or integrin‐independent mechanisms such as Piezo1‐mediated signaling. This is followed by a second “invasion” phase, ≈6 days post‐stiffening, where BM levels recover and cells actively invade. The shift in BM levels implies a potential functional transition in LN, where it goes from maintaining epithelial polarity to a permissive or even supportive factor for invasion. This biphasic behavior of LN‐332 is consistent with clinical data. Studies have reported that LN‐332 is often lost or disrupted early during breast tumor progression, particularly in ductal carcinoma in situ (DCIS) and invasive ductal carcinoma (IDC), contributing to BM breakdown and polarity loss.^[^ ^47^ ^]^ However, re‐expression of LN‐332,^[^ ^48^, ^49^ ^]^—especially its γ2 chain^[^ ^50^ ^]^—has been observed at invasive fronts of late‐stage tumors, where it supports migratory behavior and correlates with poor prognosis. Similarly, co‐elevation of FN (FN1) and β1 integrin (ITGB1) has been reported in invasive breast carcinomas, and is associated with stromal ECM remodeling, enhanced focal adhesion signaling, and reduced survival in patients.^[^ ^51^ ^]^ These clinical observations underscore the relevance of the in vitro mechanisms we describe, where Piezo1‐driven invasion emerges from dynamic β1 integrin activation and reciprocal ECM remodeling, mirroring features of malignant progression in human breast tumors. Since these phenotypic transitions are tightly coupled to matrix mechanics, it was important to resolve not only the absolute stiffness but also the dynamics of the stiffening process.

To achieve this, we combined AFM nanoindentation, which measures local Young's modulus of the surface, with PTM, which captures local viscoelastic changes within the bulk of the gel over time. While their absolute values are not directly comparable, both consistently showed an increase in stiffness after in situ crosslinking, confirming the robustness of the response. Notably, PTM revealed a ≈3.5‐fold increase within the first 6 h that plateaued thereafter, reflecting the progression of crosslinking within the bulk, whereas AFM detected an ≈100‐fold increase relative to soft gels, reflecting the higher sensitivity of local indentation to network compaction at the surface. PTM further resolved heterogeneity and temporal progression. Together, these complementary readouts with our ECM analyses demonstrate that invasion is driven by a genuine mechanical shift, raising the question of whether ECM changes stem solely from altered deposition or also from transcriptional regulation.

Previous studies have demonstrated that gene expression and matrix deposition can be uncoupled.^[^ ^35^ ^]^ In contrast, our data show a positive correlation between gene expression and deposition, suggesting that in situ stiffening likely triggers both transcriptional and post‐transcriptional cellular responses. The combined impact of these processes may underlie the magnitude of FN and LN deposition observed in our system. However, because we did not perform RT‐qPCR or bulk RNA‐sequencing in the temporal experiments shown in Figure 2, we cannot yet define the precise relationship between gene expression and acini phenotype during this highly dynamic process. This is likely to be more complex than captured here, and further investigation will be of interest in future studies.

Importantly, the invasive behaviors seen in in situ stiffened acini are phenotypically and mechanistically distinct from those observed when single MECs are grown in pre‐stiffened matrices. Unlike the complete absence of BM observed in invasive acini grown from single MECs in stiff gels, in situ stiffened acini have high levels of BM that are remodeled and localized around the invasive structures. These findings align with previous studies showing that abnormal ECM mechanics can compromise the BM barrier through protease degradation^[^ ^52^ ^]^ or mechanical forces from transformed cells,^[^ ^10^, ^53^ ^]^ The other difference we observe between the invasive phenotype in stiff versus in situ stiffened acini is the heterogeneity of phenotypes. Here, we find that in situ stiffening results in some acini being partially invasive, some completely invasive and some not responding to changes in stiffness at all which contrasts with acini grown from single MECs on stiff gels where most acini are completely invasive. We currently do not understand mechanistic basis of this heterogeneity, although it may be that the ability of normal acini to respond to stiffening is determined by the size of an acini or its cellular organization and state within the acini. We also note that large acini are modestly enriched in the fully invasive cohort and again the variation in the acini size and organization could arise from the heterogeneity in the biochemical factors present and also the local stiffness.

While we experimentally interpret these 2 phases to be distinct, it is more likely that the phases overlap, with cellular changes occurring in parallel to changes in the ECM environment. We can also assume that this initial process is independent of FN‐binding integrins such as β1 since we find negligible levels of FN in the normal mammary acini. This leads us to suggest two possible mechanisms, one that is via BM‐dependent mechanotransduction pathways or the other that is initially integrin independent. Since several studies have now shown that LN binding integrins are mechanosensitive and can respond to changes in the mechanics of the ECM,^[^ ^54^ ^]^ the BM‐dependent pathway could be through stiffness‐mediated activation of integrins such as α6β4 that eventually leads to upregulation of FN secretion. The other possible mechanism is through an upstream MSC since studies have previously shown that MSCs such as Piezo1 could also result in upregulation of FN secretion thus potentially presenting a pathway that is Integrin‐independent.^[^ ^44^ ^]^ It is important to highlight here that the mode of mechanosensing proposed would be different from mechanisms in acini grown in pre‐stiffened environment where the BM's absence would lead to potentially a LN independent mechanism.

Beyond integrin activation, in situ stiffening likely engages additional mechanotransduction circuits—including FAK–Src–RhoA/ROCK contractility,^[^ ^55^ ^]^ YAP/TAZ nuclear signaling,^[^ ^56^ ^]^ PIEZO1‐dependent Ca^2^⁺ influx,^[^ ^57^ ^]^ and force‐dependent TGF‐β activation^[^ ^58^ ^]^—that are known to be potentiated by increased matrix stiffness and to promote invadopodia maturation and invasion. Together, these pathways provide plausible routes by which in situ stiffening either activates parallel, non‐integrin mechanisms or amplifies integrin signaling to fully recapitulate the invasive phenotype. These pathways provide a mechanistic rationale for why integrin activation is sufficient to induce invasion in soft gels, whereas in situ stiffening recruits broader mechano‐signaling to achieve the full invasive state.

Lastly, our results specifically place Piezo1 activation downstream of β1 integrin‐FAK pathway and as the critical regulator of in situ stiffening mediated invasion. Activation of Piezo1 has been implicated in several cancers^[^ ^25^, ^27^, ^29^, ^32^ ^]^ but mechanisms by which Piezo1 can be activated downstream of integrins remains unknown. Studies have now shown significant cross talk between integrin and Piezo1 pathways^[^ ^29^, ^30^, ^31^, ^32^, ^33^ ^]^ such as through local changes in membrane tension in proximity of focal adhesions.^[^ ^29^ ^]^ It is also likely that Piezo1 and integrins interact with each other via more complex feedback mechanisms that regulate and amplify each other, and this needs to be probed in greater detail. While our study primarily uses pharmacological tools to probe these pathways, including PF‐573228 (FAK inhibitor), GsMTx4 (Piezo1 inhibitor), and Yoda1 (Piezo1 agonist), we note that the concentrations used are well within their reported specificity ranges. Nevertheless, we acknowledge that pharmacological agents may still exert off‐target or compensatory effects. In future studies, orthogonal validation using genetic approaches such as siRNA or CRISPR‐based knockdown will be valuable to further dissect the specificity and mechanistic interdependence of these pathways.

We speculate that in situ stiffening of acini may be more representative of tumor growth and invasion at the primary site, while single cells growing and expanding in stiff gels may reflect metastasis at secondary sites, which often originate from single or a group of metastasized cells. However, it is important to note that while this speculation provides a useful framework, the temporal dynamics of stiffening in our model differ significantly from the gradual stiffening observed in vivo. A key limitation of our model is that in situ stiffening is experimentally rapid—completed within hours—whereas ECM stiffening in vivo is gradual and often occurs over weeks or months. While this allows temporal resolution of early signaling events, it may not fully recapitulate progressive tumorigenesis. Future studies using controlled or stepwise stiffening models could further address this important temporal dimension.

A unique advantage of the in situ stiffening IPN gel system is its ability to resolve cellular responses to mechanical changes over time, a feature not achievable with stiff IPN gels. While the present study was designed to capture downstream outcomes that emerge over weeks—such as invasion, ECM deposition, and polarity—we also observed early phenotypic shifts following stiffening. Specifically, within 48 h, FN progressively accumulated, and LN decreased, indicating that matrix remodeling begins within hours of stiffening and precedes overt invasion. Although these data were limited to immunofluorescence, they highlight the temporal resolution of the system and suggest that immediate early events can be interrogated in future work. Approaches such as phospho‐FAK or pMLC immunoblotting, Ca^2^⁺ flux assays, or transcriptional profiling at early time points could provide mechanistic insight into how cells sense and respond to newly stiffened environments. Thus, even though late‐stage outcomes of in situ and persistently stiff gels converge, the capacity to capture the dynamic transitions leading to invasion represents a central novelty of the in situ platform.

In summary, our in situ stiffening system and the mechanisms discovered here following its application, have implications not only in the context of cancer and the onset of invasion but also in the context of development and the morphological changes that occur in the sculpting of complex tissues. Importantly, our findings highlight potential therapeutic strategies targeting mechanotransduction pathways involved in stiffening‐induced cellular responses. For instance, Piezo1 inhibitors could be explored to mitigate the invasive behavior of tumorigenic cells by reducing mechanosensitive calcium influx, thereby limiting their ability to breach the BM. Similarly, anti‐ β1 integrin antibodies may offer a viable approach to disrupt integrin‐mediated mechanotransduction, which is critical for cell‐ECM adhesion and signaling, ultimately reducing tumor cell invasion and metastasis. These insights underscore the relevance of targeting cellular mechanosensing mechanisms in both cancer therapy and tissue engineering applications.

## 4 Experimental Section

### Cell Culture

The following human breast epithelial cell lines were used in this study: MCF10A (RRID: CVCL_0598), MCF10DCIS.com (RRID: CVCL_5552), and MCF7 (RRID: CVCL_0031). All cell lines were generously provided by Dr. Guillaume Jacquemet (Åbo Akademi University). The cell lines were not authenticated by the authors but are widely used and well‐characterized in breast cancer research. RRIDs are provided where available to enhance reproducibility. MCF10A and MCF10DCIS.com cells were cultured in Dulbecco's Modified Eagle Medium/Nutrient Mixture F‐12 (DMEM/F12; Thermo Fisher) supplemented with 5% horse serum (Thermo Fisher), 20 ng mL^−1^ EGF (Miltenyi Biotech), 0.5 µg mL^−1^ hydrocortisone (Sigma), 100 ng mL^−1^ cholera toxin (Sigma), 10 µg mL^−1^ insulin (Sigma), and 1% penicillin/streptomycin (Thermo Fisher). MCF7 cells were cultured in DMEM (Thermo Fisher) supplemented with 10% fetal bovine serum (FBS; Thermo Fisher) and 1% penicillin/streptomycin. All cells were routinely passaged every 3–4 days using TrypLE Express Enzyme (Thermo Fisher) and maintained in a humidified incubator at 37 °C and 5% CO_2_. Mycoplasma contamination testing was not performed by the authors; however, no abnormal changes in cell morphology or proliferation were observed throughout the study. The absence of routine testing is not expected to affect the validity or interpretation of the experimental findings.

### Alginate Preparation

1% w/v unmodified sodium alginate (Pronova UP MVG, Novamatrix), rich in guluronic acid and with high molecular weight, was dissolved in ultrapure water, sterile filtered and lyophilized. Subsequently, the reconstituted alginate stock solution was prepared by dissolving the lyophilized alginate in serum free DMEM/F12 media overnight to bring the total alginate concentration to 2.5% w/v.

### IPN Gel Preparation

To prepare IPNs with final concentrations of 5 mg mL^−1^ alginate and 4.5 mg mL^−1^ BME (Cultrex Basement Membrane Extract, Type 2, Pathclear, Biotechne R&D Systems), prechilled reconstituted alginate was mixed with BME and serum‐free DMEM/F12 media in a 1.5 mL microcentrifuge tube on ice. The mixture was transferred to a 1 mL Luer Lock syringe (Fisherbrand). Separately, serum free DMEM/F12 media with or without 20 mm CaSO_4_ was loaded into a second 1 mL Luer Lock syringe for preparing stiff and soft IPNs, respectively. The two syringes were coupled using a female–female Luer‐Lock connector (Masterflex®, VWR), mixed rapidly, and immediately deposited into appropriate dishes for AFM and PTM measurements. The solutions were allowed to gelate for 1 h in an incubator at 37 °C to form stiff and soft IPN gels. If required, soft gels were in situ stiffened by incubating them in a 20 mm CaCl_2_ solution for 30 min. The in situ stiffened gels were washed thrice with phosphate buffered saline (PBS).

### Mechanical Testing

*AFM Measurements –* AFM force spectroscopy experiments were performed with a NanoWizard 4 (JPK, Bruker) mounted on a Nikon Ti‐2 inverted microscope with a x20 objective (Nikon ELWD S Plan Fluor, NA = 0.6). Measurements were performed with rectangular cantilevers with a colloidal probe of radius R = 2.25 µm (NanoAndMore, CP‐ qp‐CONT‐Au‐A). The cantilevers have a nominal resonance frequency of 30 kHz and nominal spring constant 0.1 N m^−1^. The gels were indented with a setpoint value of 0.2‐1.0 nN with a speed of 1 µm/sec and sampling rate of 1000 Hz. Measurements were performed in PBS with no magnesium and no calcium.

The analysis of the force‐distance curves is based on a previous study^[^ ^59^ ^]^ and uses the following relation between the measured force *F* and indentation *δ* for a parabolic indenter geometry

(1) $$F=\frac{4\sqrt{R_{c}}}{3}\frac{E}{1-\upsilon^{2}}\delta^{3/2}$$

where *E*, *ʋ*, and *R_c_* are the Young's modulus of the sample, the Poisson's ratio of the sample, and the effective radius of the indenter tip curvature, respectively. Poisson's ratio is assumed to be 0.5.

*PTM measurements –* PTM was used to map the dynamics of soft IPN gel stiffening upon crosslinking. PTM involves tracking the thermally driven Brownian motion of probe particles over time, using their trajectories to extract the viscoelastic properties of the surrounding material.

To image the probe particles, a widefield microscope equipped with a x60 objective lens (Olympus, numerical aperture 1.1, working distance 1.5 mm) and an sCMOS camera (Hamamatsu, ORCA Flash 4.0) was used. Soft IPN gels were prepared as previously described, with the addition of 6 µm diameter polystyrene beads to the gel solution to serve as probe particles after gelation. The prepared soft IPN gel with embedded probe particles was deposited into Ibidi μ‐Slide 4‐well glass‐bottom chambered coverslips.

Measurements were recorded over a 20‐h period, after which the soft gels were in situ stiffened by the addition of 20 mM CaCl2 at the 22‐h time point. Following a 30‐min incubation in the CaCl2 solution, the gels were rinsed three times with sterile MilliQ water.

For each rheological measurement, an individual probe particle was selected, and a small region of interest (20 × 20 µm) was imaged at a frame rate of ≈300 frames per second. A total of 300000 frames were collected per measurement. A schematic of the imaging system is shown in Figure 2, which also includes an example of a small region of interest containing a probe particle. The Brownian motion of the probe particle was tracked using a centre‐of‐mass algorithm (Figure 2). To extract the low frequency elastic modulus of the material local to the probe the analysis steps detailed in previous PTM studies,^[^ ^37^, ^38^ ^]^ The mean squared displacement, MSD, of the probe's motion is analysed, and related to the time‐dependent compliance of the material local to the probe, J(t), as follows,

(2) $$\mathrm{MSD}\left(\tau \right)=\frac{k_{B}T}{\pi a}J\left(t\right)$$

where τ is the lag‐time, k_B_ – Boltzmann constant, T – temperature, and a – the radius of the probe. The Fourier transform of the time‐dependent compliance, $\hat{J}\left(\omega \right)$, is used to determine the complex modulus, G^*^(ω) of the material, an example of which is shown in Figure 2A,

(3) $$G^{*}\left(\omega \right)=\frac{1}{i\omega \hat{J}\left(\omega \right)}$$

where the real, G’(ω), and imaginary, G″(ω), components of the complex modulus relate to the elastic and viscous response of the material respectively. In Figure 2A, we compare the elastic properties of different samples by plotting the low frequency elastic modulus (G_0_’), in other words the low frequency, long time, plateau in G’(ω).

### Acini Formation in IPN Gels

To form acini, soft and stiff IPN gels were prepared as described above, with the addition of a single‐cell suspension of MCF10A cells ((or MCF10DCIS.com or MCF7, where applicable) at a density of 35000 cells mL^−1^ to the mixture within the first syringe. After mixing the contents of both syringes, the IPN gel mixture with cell suspension was transferred to a 1.5 mL microcentrifuge tube. Subsequently, 30 µL of the soft IPN gel mixture was pipetted into each well of a 24‐well plate precoated with BME (1:5 dilution in serum‐free DMEM/F12 media) to form dome‐like drops. The plate was incubated at 37 °C for at least 1 h to allow the formation of soft IPN gels. Once the gels were set, acini culture media (DMEM/F12 media supplemented with 2% horse serum, 5 ng mL^−1^ EGF, 0.5 µg mL^−1^ hydrocortisone, 100 ng mL^−1^ cholera toxin, 10 µg mL^−1^ insulin, and 1% penicillin/streptomycin) was carefully added to the sides of the wells to prevent gel detachment. The media was replaced every 3 to 4 days throughout the culture period. Once acini had formed within the soft IPN gels by day 14, a subset of these gels was subjected to stiffening or treated with specific pharmacological agents to investigate the role of integrin or Piezo1 activation and exogenous FN on normal acini. For stiffening, the acini culture media was replaced with a 20 mM CaCl_2_ solution, and the soft IPN gels were incubated in this solution for 30 min at 37 °C. After incubation, the CaCl_2_ solution was removed, and the gels were washed three times with PBS before fresh acini culture medium, with pharmacological agents that promote or inhibit invasion in in situ stiffened conditions, if required, was added. The acini were then cultured for an additional 14 days in the in situ stiffened gels.

To form acini in soft IPN gels pre‐mixed with FN, the same procedure as described above was followed. However, a FN solution was added to the contents of the first syringe, achieving a final concentration of 20 µg mL^−1^ in the soft IPN gel mixture.

### Acini Formation in BME Gels

A single‐cell suspension of MCF10A MECs was prepared by mixing the cells with 4.5% w/v BME (diluted in serum‐free DMEM/F12 medium) in a 15 mL centrifuge tube on ice to achieve a final cell density of 35000 cells mL^−1^. The mixture was pipetted as dome‐shaped drops into each well of a 24‐well plate. The cells were cultured for 14 days to allow acini formation. On day 14, a subset of the BME gels containing fully formed acini was incubated with a 20 mm CaCl_2_ solution to investigate whether calcium induces invasive properties in normal acini within a soft microenvironment. After a 30‐min incubation at 37 °C, the BME gels were washed thrice with PBS, and fresh acini culture media was added. The acini within the BME gels were cultured for an additional 14 days.

### Real‐Time PCR

Total RNA was extracted using the RNeasy Mini Kit (Qiagen, 74104) according to the manufacturer's instructions. First‐strand cDNA was synthesized from total RNA using the iScript cDNA Synthesis Kit (Bio‐Rad, 1708891) with oligo(dT) primers (Integrated DNA Technologies). Quantitative real‐time PCR (qRT‐PCR) was performed with the iTaq Universal SYBR® Green Supermix (Bio‐Rad, 1725121) on the CFX Opus 384 Real‐Time PCR system (Bio‐Rad, 12011452). β‐actin was used as the internal reference gene, and relative expression levels were calculated using the 2^−ΔΔCt method. Each experimental group included three biological replicates; each measured in triplicate. Primer sequences used for amplification of human genes are listed in Table S1 (Supporting Information).

### Pharmacological Inhibition

Pharmacological agents were added to the acini culture media on day 14 to treat normal acini in soft gels or in situ stiffened IPN gels. The concentrations of pharmacological agents for treating normal acini in soft and in situ stiffened IPN gels are as follows:

Soft IPN gels – 0.5 mM MnCl_2_ (Sigma) for integrin activation, 10 µM Yoda1 (Tocris Bioscience, catalog number 5586) for Piezo1 activation, 20 µg mL^−1^ exogenous fibronectin (Sigma, catalog number F0895) for assessing the effects of exogenous FN

Stiff IPN gels – 5 µg mL^−1^ Anti‐Integrin β1 clone AIIB2 (Sigma catalog number MABT409) for β1 integrin inhibition, 5µM PF‐573228 (Tocris Bioscience, catalog number PZ0117) for FAK inhibition and 5 µm GsMtx4 (MedChem Express, catalog number HY‐P1410) for Piezo1 inhibition.

### Immunofluorescence Staining and Imaging

To fix IPN gel samples embedded with acini for immunofluorescence staining, 2% paraformaldehyde (Thermo Fisher) in PBS supplemented with 0.1% glutaraldehyde (Sigma) was added to the gels and incubated for 30 min at room temperature. The gels were washed three times with PBS and then permeabilized using 0.15% Triton X‐100 (Fisher Scientific) for 25 min. Afterward, they were washed again three times with PBS and incubated overnight in a blocking buffer consisting of 3% BSA solution in PBS.

The gels were subsequently incubated with primary antibodies (1:400), diluted in 1% BSA and 0.3% Triton X‐100 in PBS, overnight at 4 °C. After washing three times with PBS, the gels were incubated in a solution containing fluorescently conjugated secondary antibodies, Alexa Fluor 488–phalloidin (1:400, Thermo Fisher), and Hoechst 33342 solution (1:4000) for 90 min at room temperature, followed by three PBS washes. The gels were mounted with ProLong Glass Antifade Mountant and sandwiched between two round coverslips.

Images of the gel samples on coverslips were acquired using a Nikon Confocal A1RHD microscope equipped with 405‐, 488‐, 561‐, and 640‐nm laser lines and GaAsP PMTs, using x10 and x20 objectives with numerical apertures of 0.45 and 0.8, respectively.

Primary antibodies used included anti‐fibronectin (from rabbit, Sigma, catalog number F3648, 1:400), anti‐laminin‐5 (γ2 chain, clone D4B5, from mouse, Sigma, catalog number MAB19562, 1:400), anti‐β1 integrin (from mouse, Sigma, catalog number MAB2247, 1:400), anti‐α3 integrin (clone P1B5, from mouse, Sigma, catalog number MAB1952Z) and anti‐β4 integrin (clone 439‐9B, from rat, Thermo Fisher, catalog number 14‐1049‐82, 1:400). Secondary antibodies used were Alexa Fluor 568 goat anti‐mouse IgG (Thermo Fisher, 1:400), Alexa Fluor 568 goat anti‐rabbit IgG (Thermo Fisher, 1:400), Alexa Fluor 647 goat anti‐mouse IgG (Thermo Fisher, 1:400), and Alexa Fluor 647 goat anti‐rabbit IgG (Thermo Fisher, 1:400).

### Image Analysis and Quantification of Acini Phenotypical Parameters

All image analyses, including the quantification of acini area, acini circularity, and mean intensities of FN and LN expressed by acini, were performed in ImageJ. x10 and x20 image sequences acquired with the confocal microscope were converted into Z‐projection (maximum intensity projection) images and background‐subtracted.

Acini images from at least three biological replicates per experimental condition were used for quantification. Acini were manually categorized as partially invasive, completely invasive, normal, or non‐invasive based on descriptions provided (see results). Acini area and circularity were measured by manually tracing the outline of the F‐actin/Phalloidin channel of Z‐projected acini images in ImageJ.

The mean intensities of FN, LN, β1 integrin, and β4 integrin were calculated by measuring the mean pixel intensity of their respective staining within the same manually drawn regions of interest used for area and circularity measurements. Normalized FN and LN enrichment were calculated using the formula:

Normalized FN or LN Enrichment = Mean Intensity of FN or LN of an acinus/ Sum of Mean Intensities of FN and LN of the same acinus

A Fire LUT was applied to Z‐stacked, background‐subtracted FN and LN staining images to visualize ECM remodeling in representative acini across all figures.

### Statistical Analysis

All data were analyzed using GraphPad Prism version 10 (GraphPad Software, Boston, Massachusetts, USA, www.graphpad.com). The specific number of acini per biological replicate for each experimental condition is indicated as *n* in the respective figure legends. Non‐normally distributed data were analyzed using a Mann‐Whitney U test. Scatter plots with bars, stacked bar plots, and dot plots were used for data display, with horizontal lines representing the mean.

Statistical tests used for each experiment are displayed in the corresponding figure legends. Asterisks indicate statistical significance, with the following *p*‐value ranges: ^****^ *p* < 0.0001, ^***^ *p* < 0.001, ^**^ *p* < 0.01, ^*^ *p* < 0.05, ns, not significant.

## Conflict of Interest

The authors declare no conflict of interest.

## Author Contributions

K.S. and V.S. performed conceptualization; K.S. and V.S. performed methodology; K.S., A.K., E.J.P., C.M., A.C.S., A.J.W., and V.S. performed material characterization and analysis; K.S. and Z.F. performed formal analysis; K.S. and Z.F. performed investigation; K.S. and V.S. wrote–original draft; K.S., A.K., E.J.P., C.M., P.N., A.C.S., A.J.W., and V.S. wrote–review and edited; K.S. and V.S. performed visualization; V.S. performed Funding acquisition; V.S. performed supervision.

## Supporting information



Supporting Information

## Data Availability

The data that support the findings of this study are available from the corresponding author upon reasonable request.
