# Supplementary material for: Integrin‐Piezo1 Axis Drives ECM Remodeling and Invasion of 3D Breast Epithelium
Source: Adv Sci (Weinh). 2025 Oct 13;13(5):e09932. doi: 10.1002/advs.202509932 (PMC12849879; doi:10.1002/advs.202509932)
Supplement: Supplementary file 1 — Supporting Information [file ADVS-13-e09932-s001.pdf]

Supplementary Figure 1

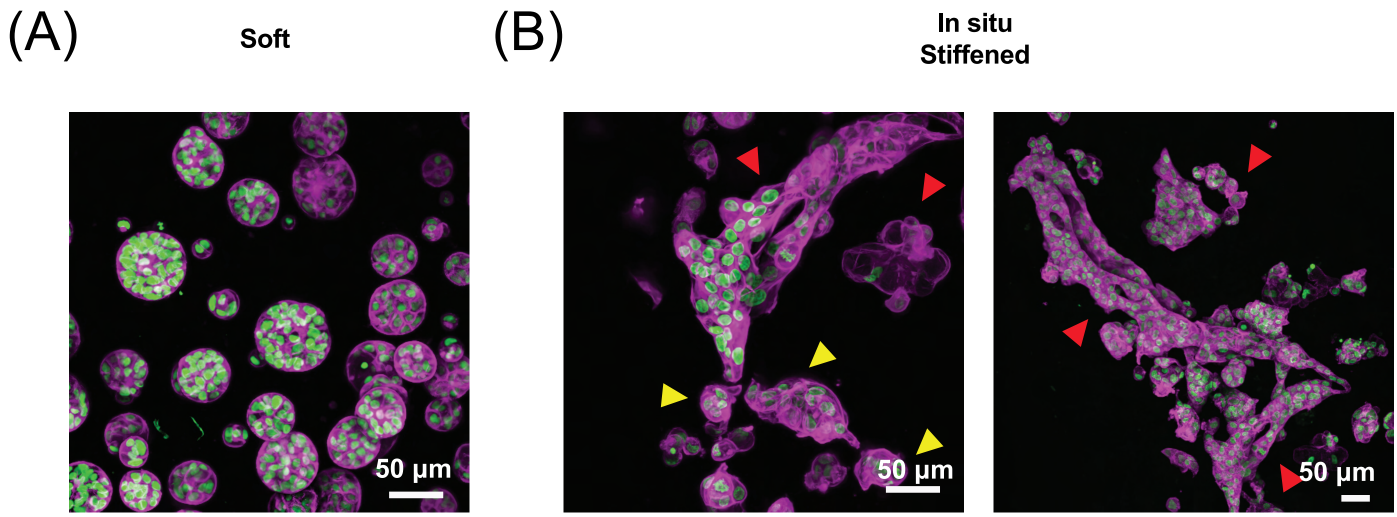

Supplementary Figure 1. Diverse acini morphologies and invasion patterns in soft and in situ stiffened IPN gels

Z-projected confocal images showing nuclei (green) and F-actin (magenta) illustrate the morphological diversity of acini on day 28 in (A) soft and (B) in situ stiffened IPN gels. Soft gels predominantly contained normal mature acini, whereas in-situ stiffened gels displayed a mix of normal, partially invasive (yellow triangles), and completely invasive acini (red triangles). Scale bar: 50 µm.

Supplementary Figure 2

(A)

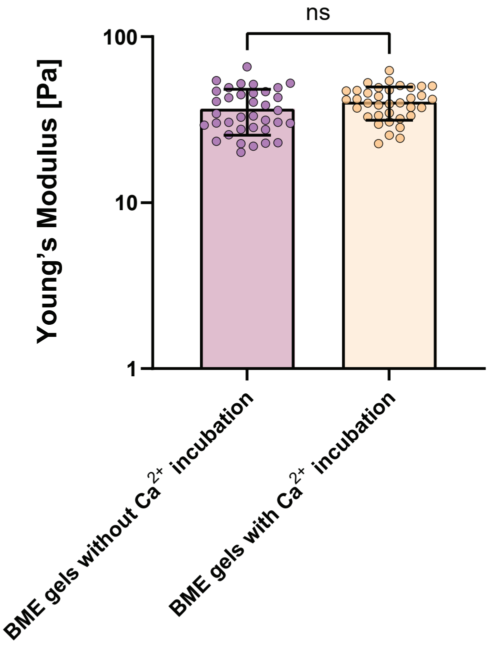

(B)

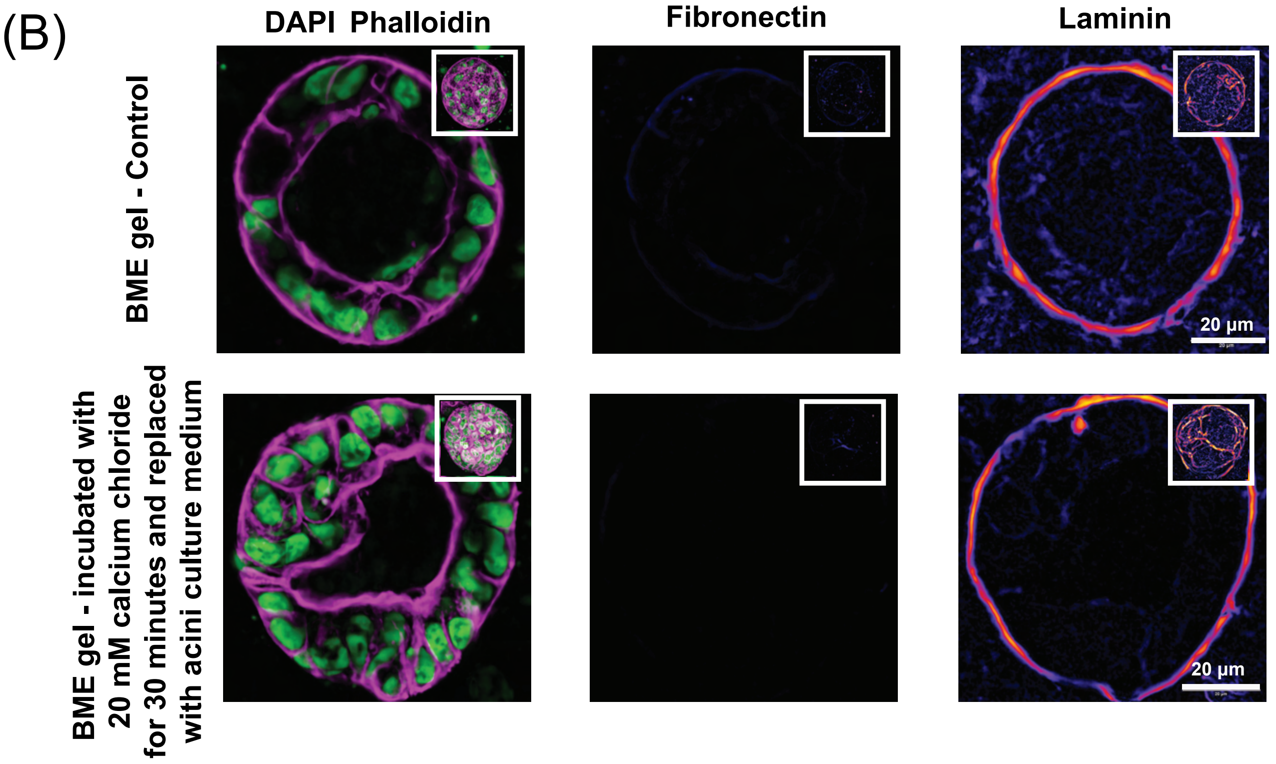

Supplementary Figure 2. Impact of calcium ions on acini morphology and invasion in BME gels

(A) AFM measurements of the Young's modulus of BME gels incubated without and with 20 mM calcium chloride (immediate 30-minute incubation after the gel formation followed by a three-time PBS wash). Stiffnesses were measured after 24 hours after the formation of BME gels. (n = 2 different experiments per condition, with 2 × 3 - point measurements performed on three distinct regions in each experiment).

(B) Confocal images showing nuclei (green), F-actin (magenta), and FN and LN (with fire LUT applied to highlight FN and LN levels within the acini) staining of acini cultured in BME gels, without and with the incubation of 20 mM $CaCl_2$. Single-cell suspensions of MECs were encapsulated in BME gels without alginate and cultured for two weeks. After acini formation, a subset of the BME gels was incubated with 20 mM $CaCl_2$ for 30 minutes. The $CaCl_2$-treated gels were then washed three times with PBS before acini culture medium was added for a subsequent two-week culture period. A single Z-plane (middle plane of the stack) images of acini cultured in both the conditions are displayed to highlight lumen formation and retention (Z-stacked images for the same acini are shown in the inset). Notably, the presence of calcium ions alone did not induce invasive phenotypes in normal mammary acini, as evidenced by the retention of non-invasive acinar structures. Scale bar: 20 µm.

Supplementary Figure 3

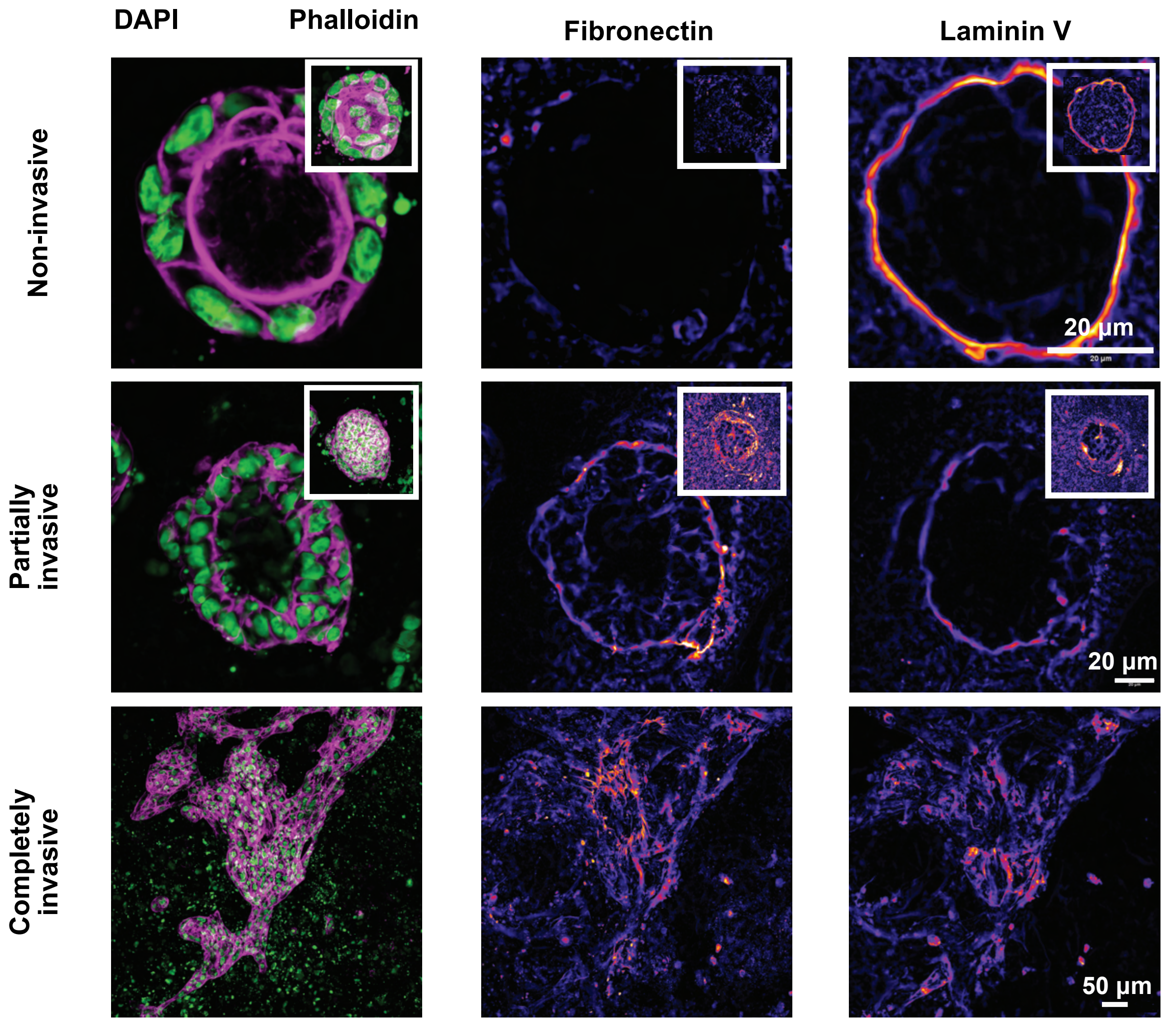

Supplementary Figure 3. In situ stiffening of soft IPN gels induces distinct invasive phenotypes and ECM remodeling in mammary acini

Confocal images showing representative acini with varying levels of responsiveness to in situ stiffening of soft IPN gels. Each example represents a distinct invasive phenotype: non-invasive, partially invasive, and completely invasive structures. The images highlight differences in nuclei (green) and F-actin (magenta) morphologies, along with FN and LN expressions (fire LUT applied to emphasize their enrichment) within each acinus. Scale bar: 20 μm for non-invasive and partially invasive acini and 50 μm for completely invasive acini.

Supplementary Figure 4

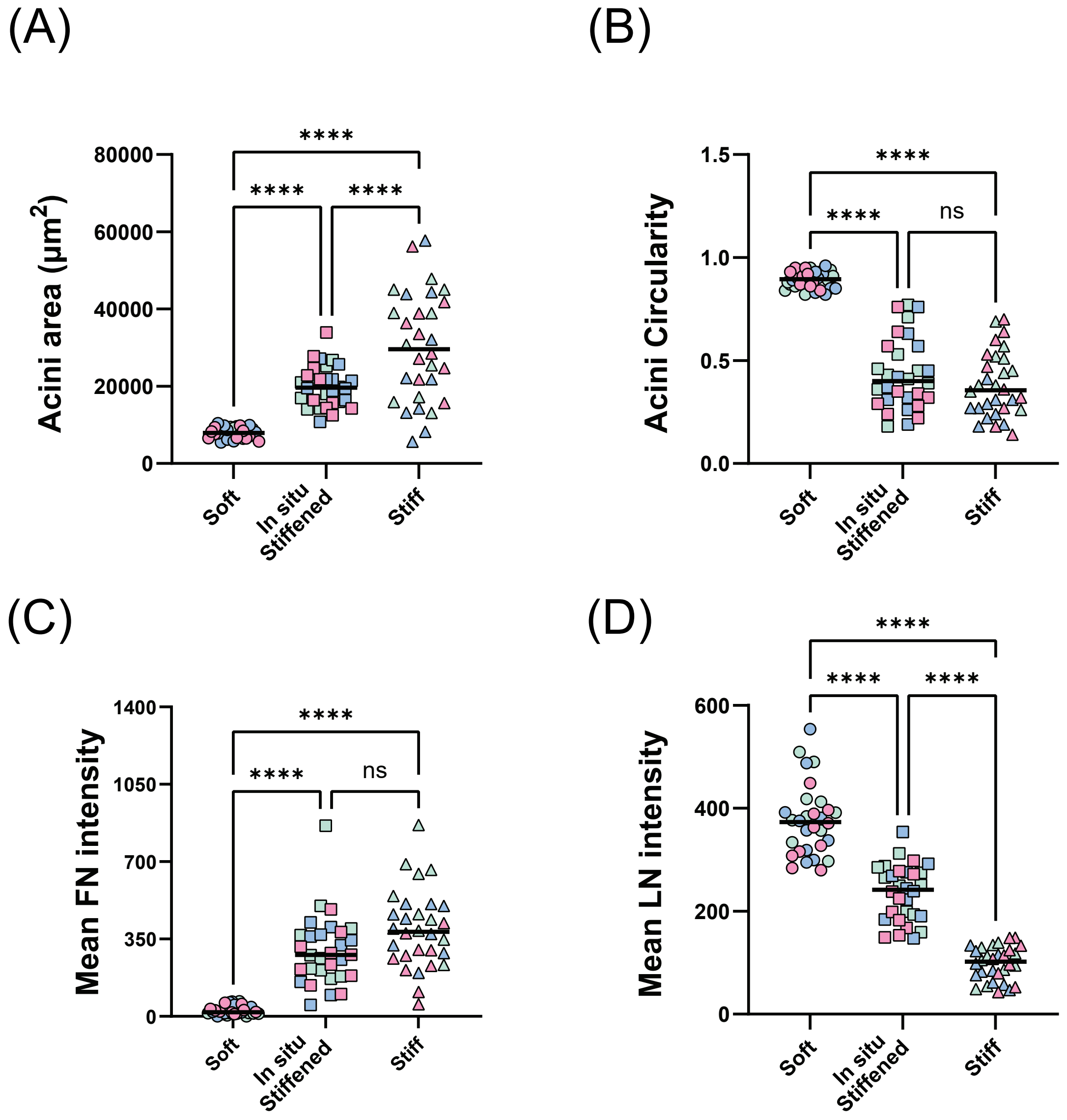

Supplementary Figure 4. Quantification of acini phenotypical parameters in soft, in situ stiffened, and stiff IPN gels.

*(A)-(D) Quantification of (A) acini area, (B) acini circularity and mean intensities of (C) FN and (D) LN expression in acini cultured within soft, in situ stiffened, and stiff IPN gels. Each color on the plot represents data from different biological replicates (n = 30 acini from 3 different experiments, with 10 acini per experiment for acini area, circularity, and FN and LN enrichment; Ordinary one-way ANOVA test for acini area, acini circularity and mean LN intensity quantifications, Kruskal-Wallis test for mean FN intensity quantification, ****p < 0.0001, ns=non-significant).*

Supplementary Figure 5

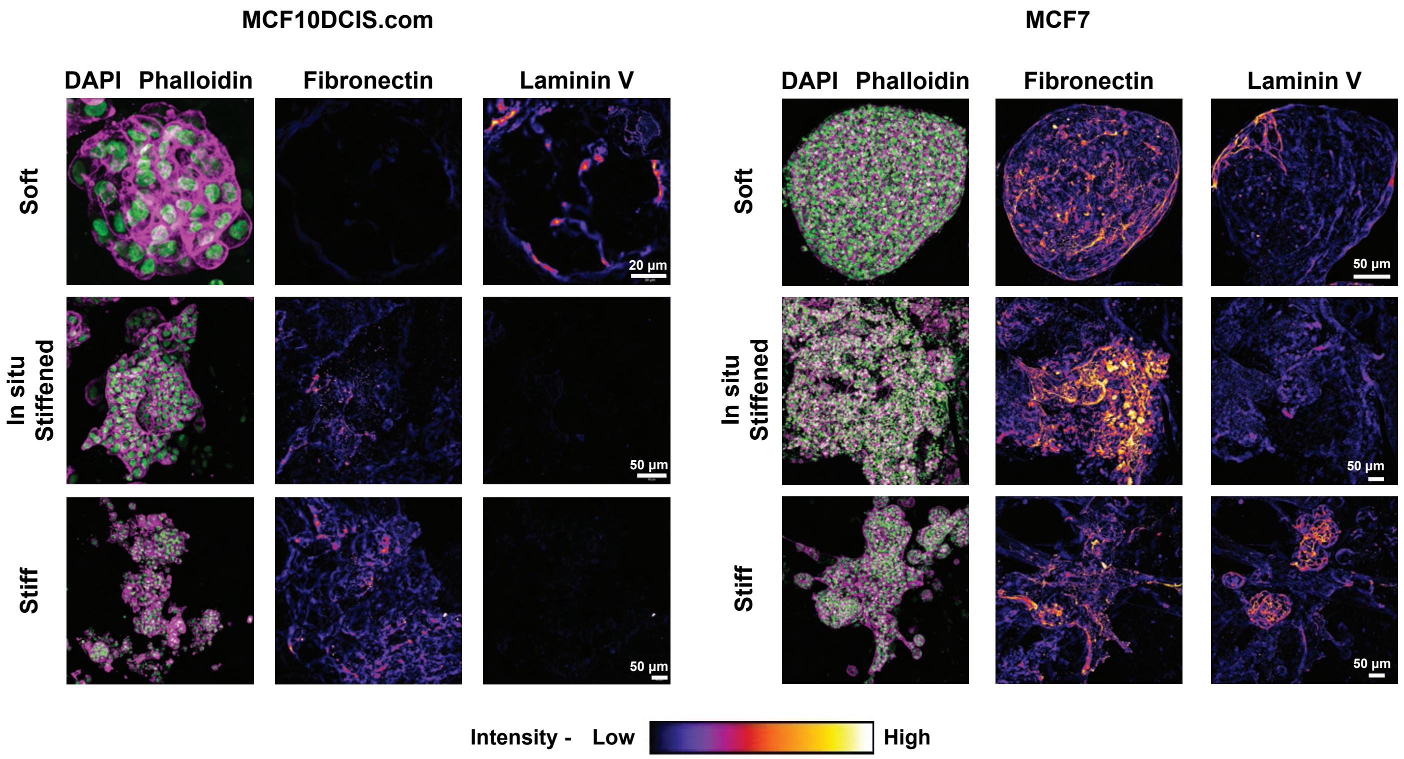

Supplementary Figure 5. In situ stiffening of soft IPN gels also induces invasion and ECM remodeling in tumorigenic epithelial cells (MCF10DCIS.com and MCF7)

Z-projected confocal images showing nuclei (green), F-actin (magenta), and FN and LN staining (fire LUT applied to highlight FN and LN levels within the acini) of MCF10DCIS.com and MCF7 acini formed in soft, in situ stiffened and stiff IPN gels. Scale bars: 20 µm for soft; 50 µm for in situ stiffened and stiff.

Supplementary Figure 6

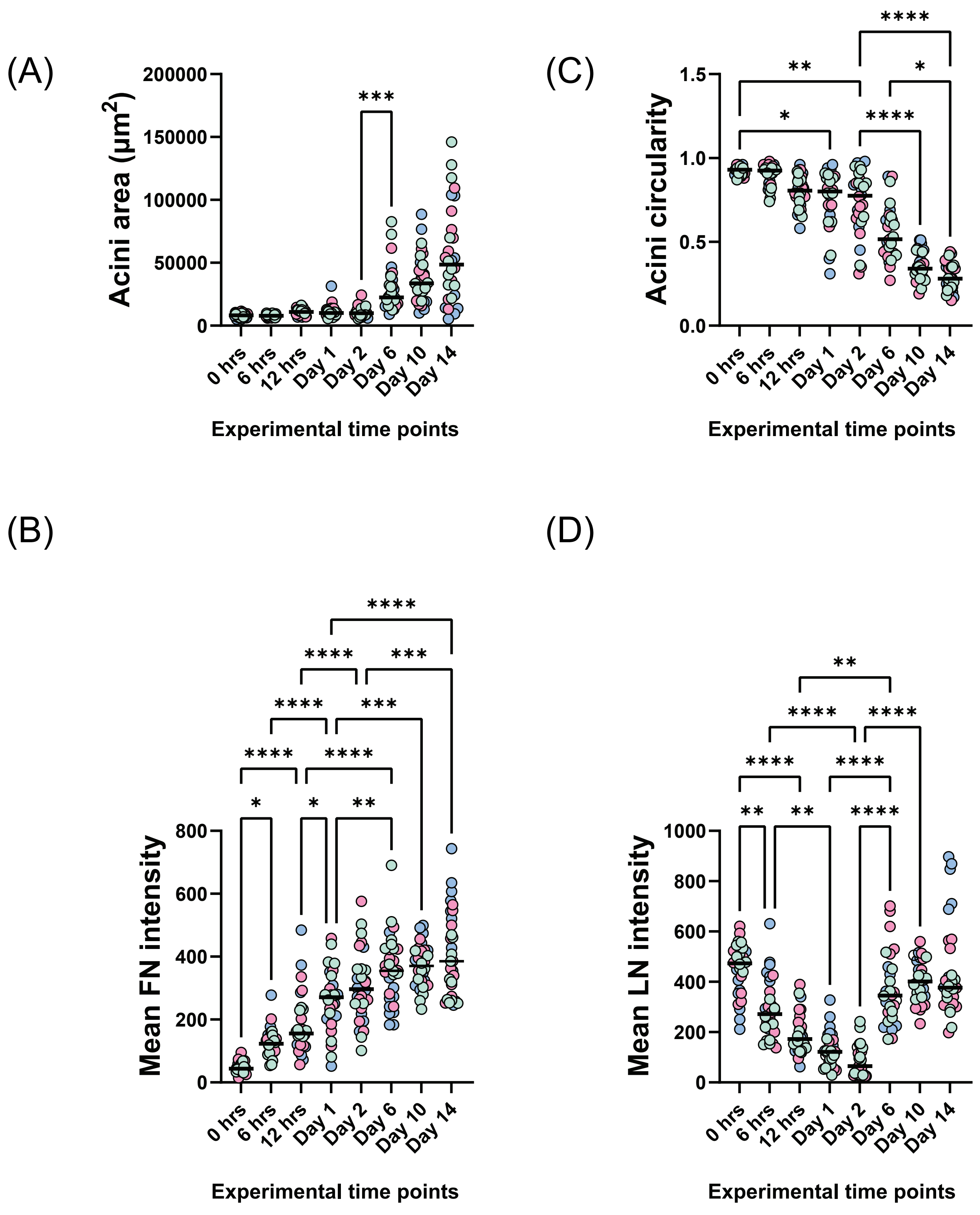

Supplementary Figure 6. Quantification of phenotypical parameters of acini in in situ stiffened IPN gels at different time points post stiffness modulation.
*(A)-(D) Quantification of (A) acini area, (B) acini circularity, and mean intensities of (C) FN and (D) LN expressions in acini in in situ stiffened IPN gels at different time points post the stiffening process. (n = 30 acini from 3 different experiments, with 10 acini per experiment for all quantifications; Kruskal-Wallis test, *p < 0.05, **p < 0.01, ***p < 0.001, ****p < 0.0001).*

Supplementary Figure 7

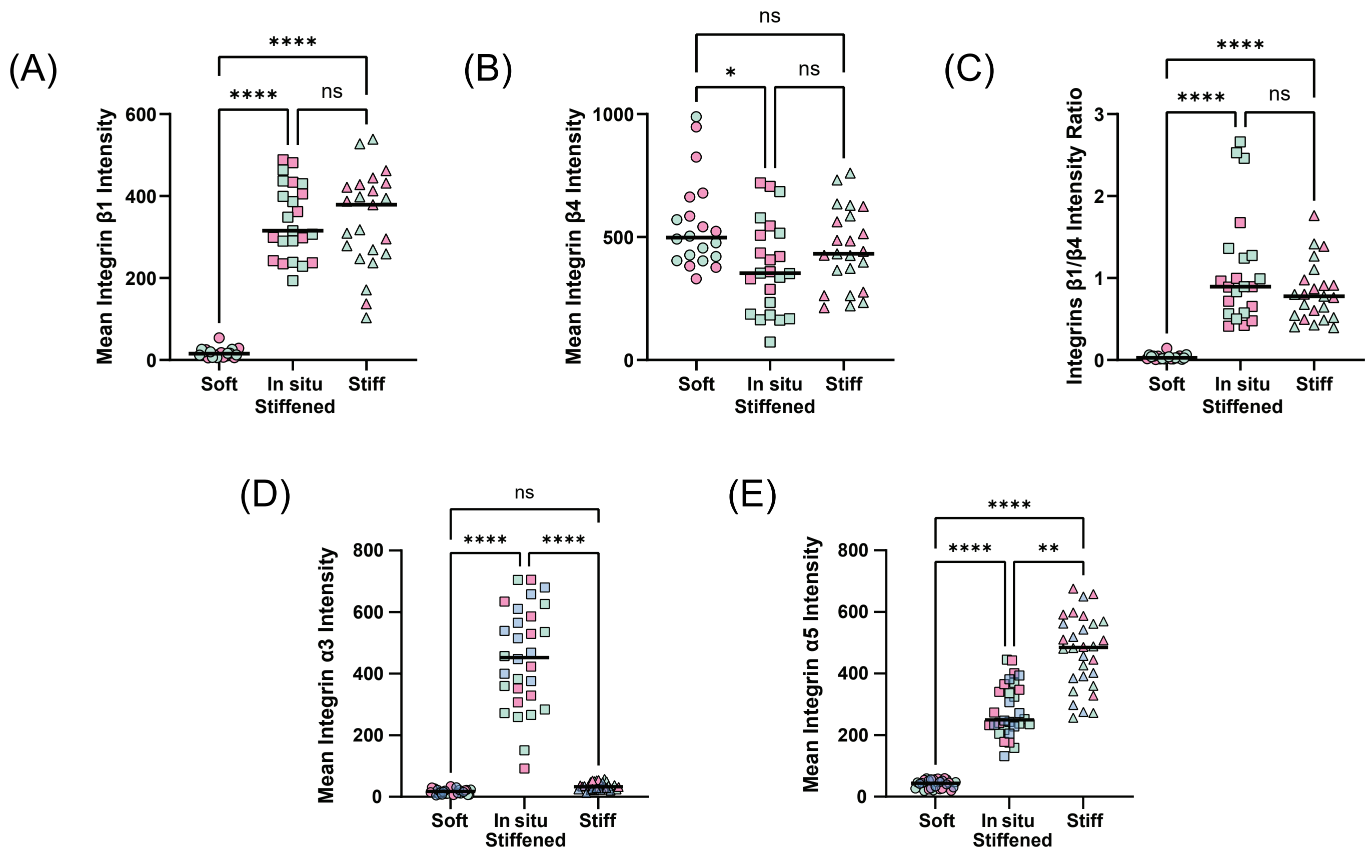

Supplementary Figure 7. Quantification of mean intenstities of different integrin subtypes in acini cultured under different experimental conditions demonstrating the role of the integrin mediated ECM remodeling.

*(A)-(C) Quantification of mean intensities of (A) β1 integrin and (B) β4 integrin expressions and (C) mean intensity ratio between β1 and β4 integrins in acini cultured within soft, in situ stiffened, and stiff IPN gels. Each color on the plot represents data from different biological replicates (n = 20 acini from 2 different experiments, with 10 acini per experiment for all quantifications; Kruskal-Wallis test, *p < 0.05, ****p < 0.0001, ns=non-significant).(D)-(E) Quantification of mean intensities of (A) α3 integrin and (B) α5 integrin expressions in acini cultured within soft, in situ stiffened, and stiff IPN gels. Each color on the plot represents data from different biological replicates (n = 30 acini from 3 different experiments, with 10 acini per experiment for all quantifications; Ordinary one-way ANOVA for α3 integrin and Kruskal-Wallis test for α5 integrin quantifications, **p < 0.01, ****p < 0.0001, ns=non-significant).*

Supplementary Figure 8

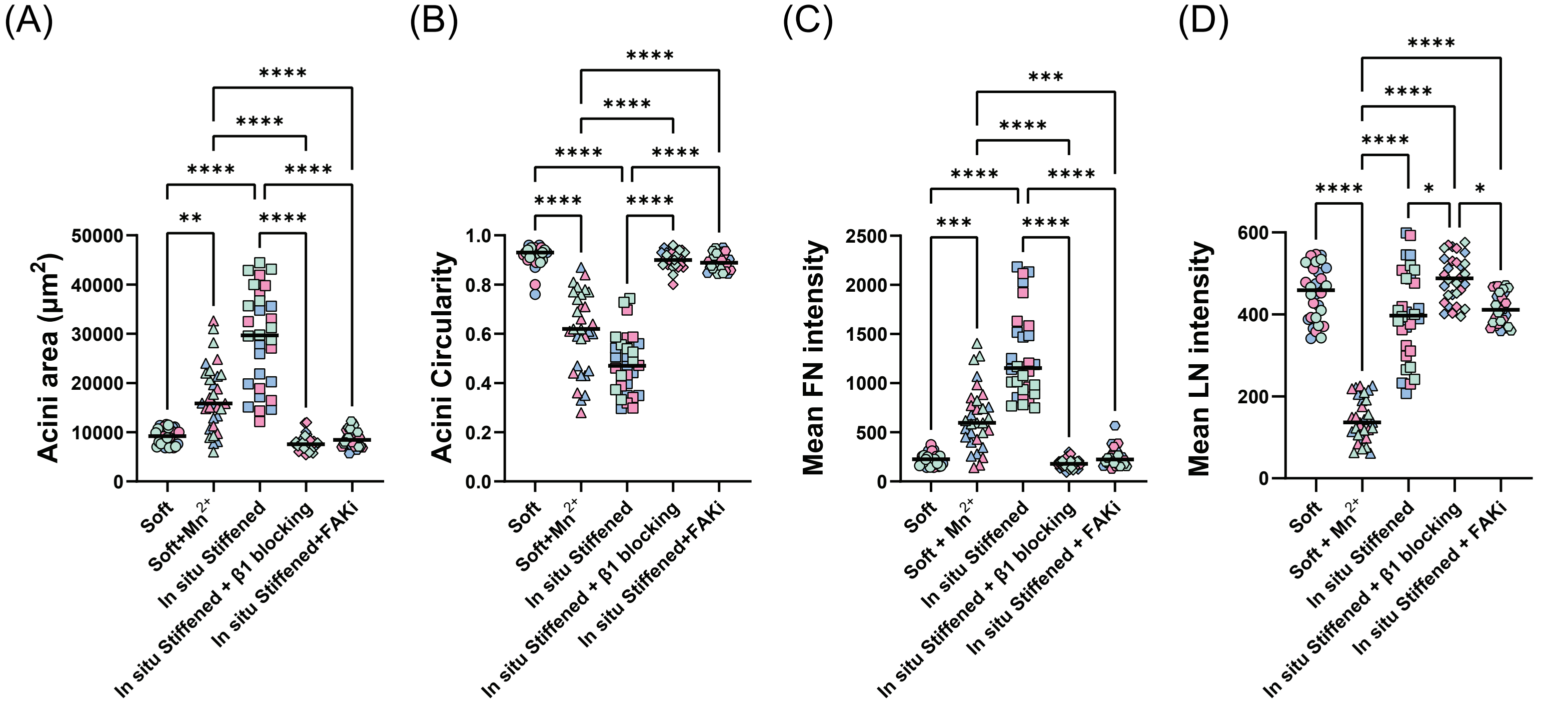

Supplementary Figure 8. Quantification of phenotypical parameters of acini cultured in different experimental conditions to demonstrate the role of the β1 Integrin-FAK signaling axis in mediating the invasive phenotype in acini post stiffening

*(A)-(D) Quantification of (AD) acini area, (B) acini circularity, and mean intensities of (C) FN and (D) LN expressions in acini cultured in soft, soft + Mn^{2+}, in situ stiffened, in situ stiffened + β1 blocking and in situ stiffened + FAKi conditions. Each color on the plot represents data from different biological replicates (n=30 acini from 3 different experiments, with 10 acini per experiment for for all quantifications; Kruskal-Wallis test, *p < 0.05, **p < 0.01, ***p < 0.001, ****p < 0.0001).*

Supplementary Figure 9

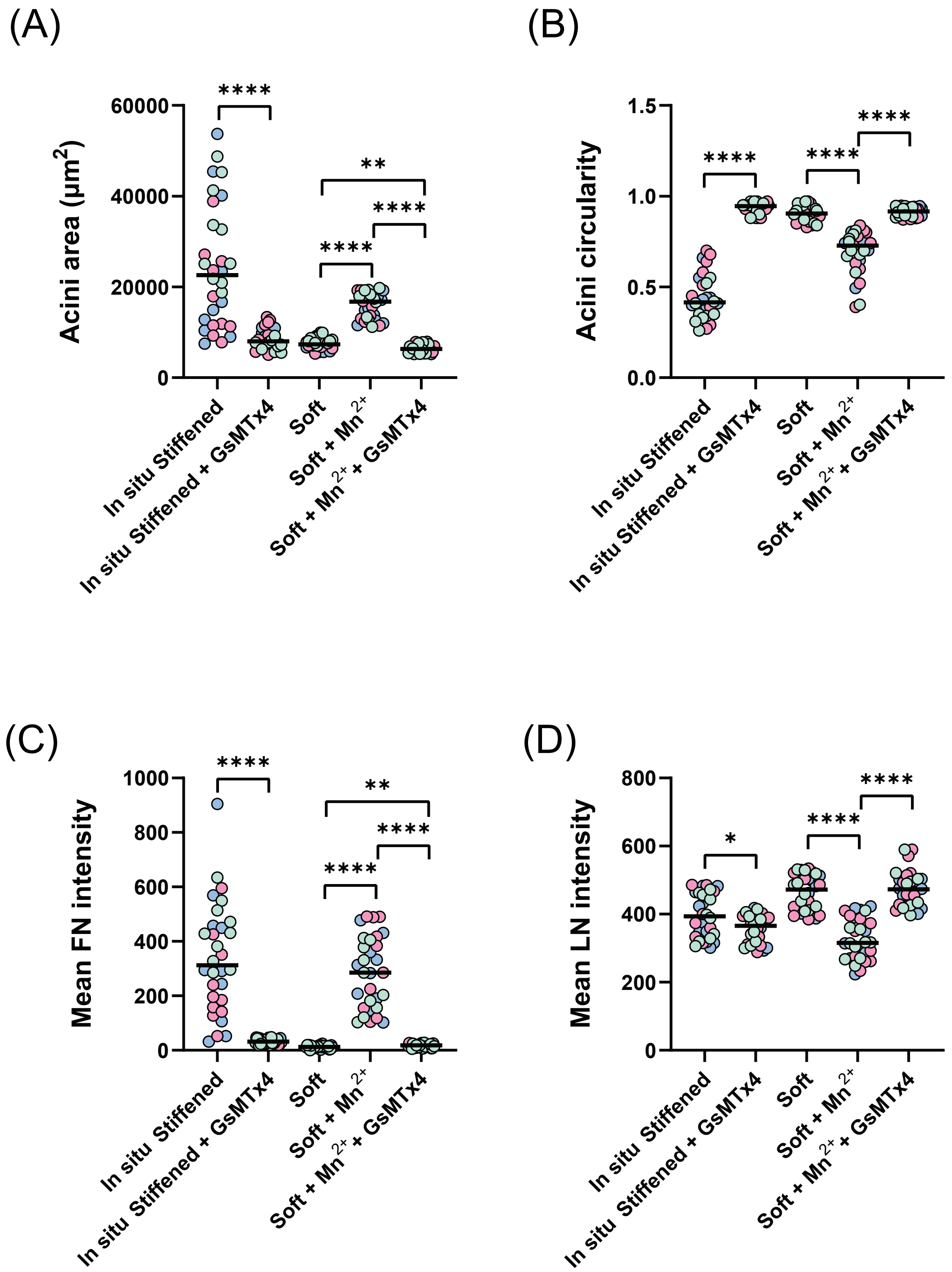

Supplementary Figure 9. Quantification of phenotypical parameters of acini cultured in different experimental conditions to demonstrate the role of MSC activity in mediating the invasive phenotype *(A)-(D) Quantification of (A) acini area, (B) acini circularity, and mean intensities of (C) FN and (D) LN expressions in acini cultured in in situ stiffened, in situ stiffened + GsMTx4, soft, soft + Mn^{2+}, soft + Mn^{2+} + GsMTx4 conditions. Each color on the plot represents data from different biological replicates (n=30 acini from 3 different experiments, with 10 acini per experiment for, all quantifications, Kruskal-Wallis test, *p < 0.05, **p < 0.01, ****p < 0.0001).*

Supplementary Figure 10

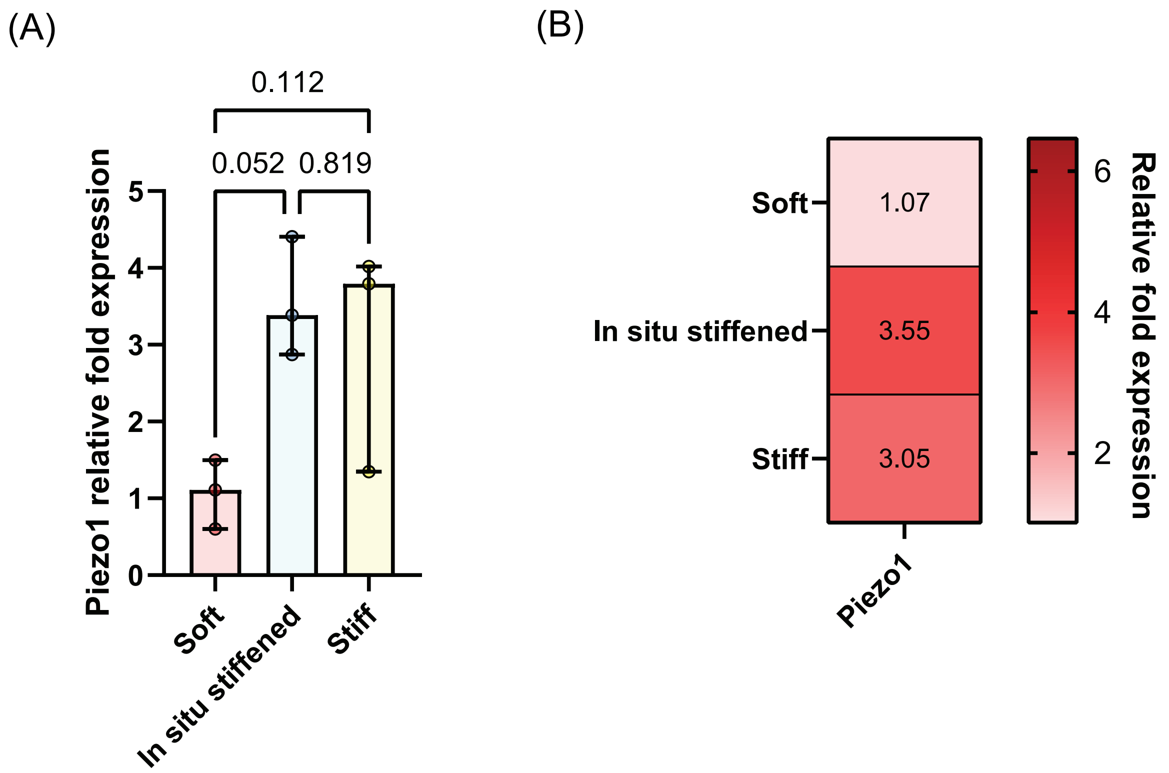

Supplementary Figure 10. Quantification of relative gene expression of Piezo1 in acini cultured in different experimental conditions *(A) Quantification and (B) heatmap of relative gene expression of Piezo1 in acini embedded in soft, in situ stiffened and stiff IPN gels (n=3 gels; Ordinary one-way ANOVA test). All changes are reported relative to β-actin expression in acini grown in respective IPN gels.*

Supplementary Figure 11

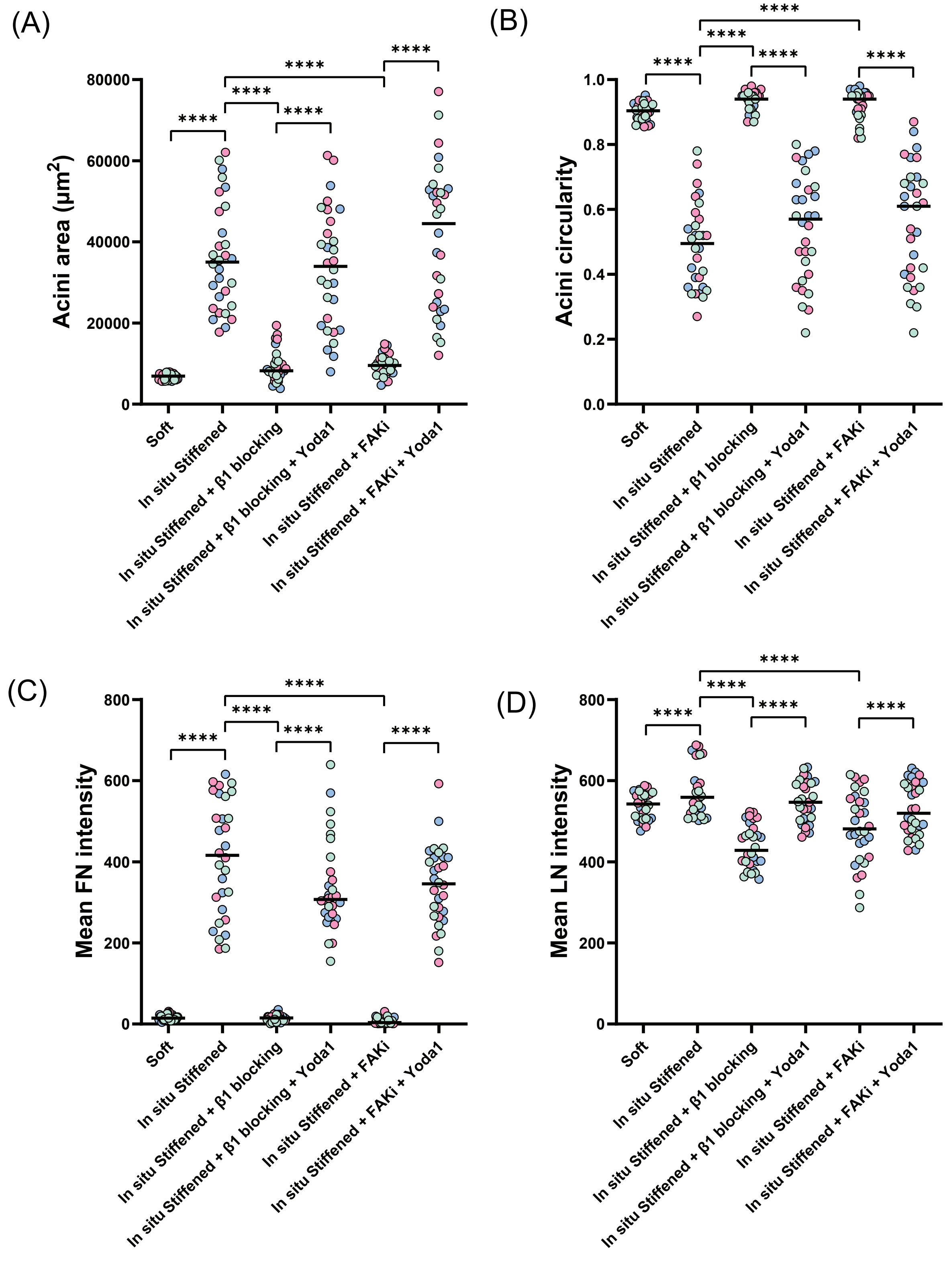

Supplementary Figure 11. Quantification of phenotypical parameters of acini cultured in different experimental conditions to demonstrate that Piezo1 functions downstream of the β1 Integrin–FAK signaling axis

*(A)-(D) Quantification of (A) acini area, (B) acini circularity, and mean intensities of (C) FN and (D) LN expressions in acini cultured in soft, soft + Mn^{2+}, in situ stiffened, in situ stiffened + β1 blocking, in situ stiffened + β1 blocking + Yoda1, in situ stiffened + FAKi conditions and in situ stiffened + FAKi + Yoda1 conditions. Each color on the plot represents data from different biological replicates (n=30 acini from 3 different experiments, with 10 acini per experiment for all quantifications, Kruskal-Wallis test, ****p < 0.0001).*

F_FN1	AGG AAG CCG AGG TTT TAA CTG
R_FN1	AGG ACG CTC ATA AGT GTC ACC
F_LAMA3	TGC TAA CAG TAT CCG GGA TTC T
R_LAMA3	TCT TGG TTC AAG CCA TTT GCC
F_LAMB3	GCA GCC TCA CAA CTA CTA CAG
R_LAMB3	CCA GGT CTT ACC GAA GTC TGA
F_LAMC2	TGG AGA ACG CTG TGA TAG GTG
R_LAMC2	CAG GAG ACC CAT TTC GTT GGA
F_betaActin	CAT GTA CGT TGC TAT CCA GGC
R_betaActin	CTC CTT AAT GTC ACG CAC GAT
F_PIEZO1	GGA CTC TCG CTG GTC TAC CT
R_PIEZO1	GGG CAC AAT ATG CAG GCA GA

Supplementary Table 1. Primer sequences used for amplification of human genes.
The listed primer sequences were used to quantify relative fold changes in acini cultured within soft, in situ stiffened, and stiff IPN gels: FN1 (fibronectin), LAMA3, LAMB3, LAMC2 (laminin-332/laminin-V), Piezo1, and β-actin.
